# The role of dietary patterns on epigenetic and inflammatory aging based on the INSPIRE-T study

**DOI:** 10.1038/s43856-026-01643-1

**Published:** 2026-05-13

**Authors:** Natasha Grande de França, Yves Rolland, Sophie Guyonnet, Paul Bensadoun, Jean-Marc Lemaitre, Bruno Vellas, Philipe de Souto Barreto

**Affiliations:** 1 IHU HealthAge, Toulouse, France; 2 Institut du Vieillissement, Gérontopôle de Toulouse, Centre Hospitalo-Universitaire (CHU) de Toulouse, Toulouse, France; 3 CERPOP, UPS/Inserm, Toulouse, France; 4 INSERM IRMB UMR1183, Hôpital Saint Eloi, Université de Montpellier, Montpellier, France; 5 CHU de Toulouse, Toulouse, France; 6 Institut RESTORE - UMR 1301 - Inserm/5070-CNRS/EFS, Université de Toulouse, Toulouse, France; 7 I2MC, Institut des Maladies Métaboliques et Cardiovasculaires, Inserm UMR 1297, Université de Toulouse, Toulouse, France; 8 Infinity Lab, INSERM UMR 1291, CNRS UMR 5051, Université de Toulouse, Toulouse, France; 9 Tonic Lab, ToNIC, Toulouse NeuroImaging Centre, UMR 1214,INSERM, Université de Toulouse, Toulouse, France; 10 CRCA/CBI lab, Centre de Recherches sur la Cognition Animale (UMR 5169), Centre de Biologie Intégrative, CNRS, Université de Toulouse - Bât 4R4, Toulouse cedex 09, France; 11 IRSD Lab, Inserm—ENVT—INRAE, Université de Toulouse, Toulouse, France; 12 CRCT Lab, CNRS UMR 5071, Université de Toulouse, Toulouse, France

**Keywords:** Prognostic markers, Lifestyle modification

## Abstract

**Background:**

Diet is a modifiable lifestyle factor that may modify biological aging. However, its relationship with biomarkers of biological aging is scarce or divergent. Thus, we aimed to investigate the association between dietary patterns and epigenetic and inflammatory age acceleration.

**Methods:**

In this cross-sectional study with 764 adults (62% female; 20-100 years), participants in the INSPIRE-T observational cohort (France), we used linear regression models to associate dietary patterns (data-driven and as adherence scores to well-established diets) with biological age acceleration estimated by using epigenetic clocks (Horvath’s, Hannum’s, PhenoAge, and GrimAge) and the inflammatory clock (iAge). We further explored the moderating effect of sex and age groups (20-44, 45-64, ≥65), and the mediating role of body fat, measured by DXA.

**Results:**

Here, we show that a 10-point increase in the Dietary Approaches to Stop Hypertension diet (DASH) score is associated with a 1.7-year lower PhenoAge acceleration, with 23% of this association being explained by android fat. Every one-unit increase in the “Plant-based” dietary pattern scores is marginally associated with 1.1-year lower PhenoAge acceleration, with total body fat accounting for 26% of this association. In the latter, the association seems more robust in older males. No consistent associations are observed for other dietary patterns, Horvath’s and Hannum’s clock, GrimAge, or iAge.

**Conclusions:**

Greater scores in aDASH and “Plant-based” dietary patterns are associated with lower epigenetic age acceleration through reduced body fat, with a partial moderating role of sex and age.

## Abstract (web summary)

Grande de França et al. associated different dietary patterns with biological age acceleration estimated by epigenetic clocks and the inflammatory clock. Greater scores in the DASH diet and the“Plant-based” dietary pattern are associated with lower epigenetic age acceleration through reduced body fat, with a partial moderating role of sex and age.

## Plain language summary

As people live longer, it becomes more important to understand how daily habits such as diet affect the way we age. This study examined whether different dietary patterns are linked to faster or slower biological aging in 764 French adults aged 20 to 100 years. Participants’ food intake was compared with measures of biological aging, assessed through blood measures, and body fat. People who more closely followed a DASH-style diet or a plant-based diet had signs of slower biological aging. Part of this benefit was explained by lower levels of body fat. The link with a plant-based diet was especially clear in older men. These findings suggest that eating more fruits, vegetables, whole grains, legumes, and low-fat dairy may help slow biological aging.

## Introduction

We age chronologically at the same rate, but not biologically. Among older adults at the same chronological age, those with accelerated biological aging are more likely to develop age-related diseases, such as type II diabetes and cancer, have poorer cognitive functioning, and display more sensory-motor difficulties^1^. Thus, the geroscience approach proposes that many age-related conditions could be prevented by slowing biological aging itself^2^.

As aging involves cumulative epigenetic alterations to the genome and a low-grade yet continuous inflammatory state, measures of biological age include epigenetic and inflammatory markers^2^. DNA methylation is the most investigated epigenetic alteration used as the basis for the development of tools called “epigenetic clocks”^3^. Some epigenetic clocks include the first-generation Hannum’s clock^4^ and Horvath’s clock^5^ (chronological age-trained), and second-generation clocks, PhenoAge^6^ (based on clinical and biological parameters predictive of morbidity) and GrimAge^7^ (mortality-trained clock). A metric for systemic chronic inflammation was proposed by Sayed et al.–the “inflammatory clock of aging” (iAge)^8^.

Diet is a modifiable risk factor for many chronic metabolic diseases, making it a potential key influencer of biological aging^9^. Cohort studies suggest that pro-inflammatory and pro-oxidative diets, as well as a higher intake of ultra-processed foods, are associated with accelerated biological aging^9–11^. On the other hand, healthier dietary patterns (DPs), such as the Mediterranean diet, have been associated with delayed biological aging^10,12–15^. Although these studies provide relevant information on the diet-biological age axis, most of them were conducted with the same cohort, considered a single marker of biological age, and tested only adherence to pre-defined DP, which may restrict the complexity of food intake in different contexts. Furthermore, few have assessed differences between sexes or ages, which is relevant since males age biologically faster than females^16^, and dietary habits change and may have diverse impacts depending on the stage of life^17,18^. Finally, diet influences body weight, which has been positively associated with biological age^19^. Moreover, DNA methylation levels at CpG sites within genes involved in longevity-regulating pathways have shown associations with body mass index (BMI), with notable differences between younger and older individuals^20^. However, weight and BMI are limited proxies for body composition, highlighting the need for more precise measures. Current literature lacks the relationship between diet and the inflammatory clock.

Considering the current evidence, our main hypothesis was that healthier DPs are associated with decelerated biological aging, with this association being mediated by body fat. We further hypothesized that the relationship between DPs and biological aging behaves differently between men and women, and between younger and older adults. Thus, we explored the relationship between DPs extracted by two different approaches, *a posteriori* (data-driven DPs) and a priori (adherence to the Mediterranean diet and the Dietary Approaches to Stop Hypertension (DASH)- diet), and several measures of biological age acceleration by using epigenetic and inflammatory clocks. We further explored whether the associations were moderated by sex and chronological age, and mediated by body fat.

Greater adherence to the DASH diet and higher conformity to a “Plant-based” dietary pattern are associated with lower biological age acceleration, partially explained by reduced body fat. There is a moderating role of sex and age, with more robust evidence in older males.

## Methods

### Participants and study design

**Fig. 1:**
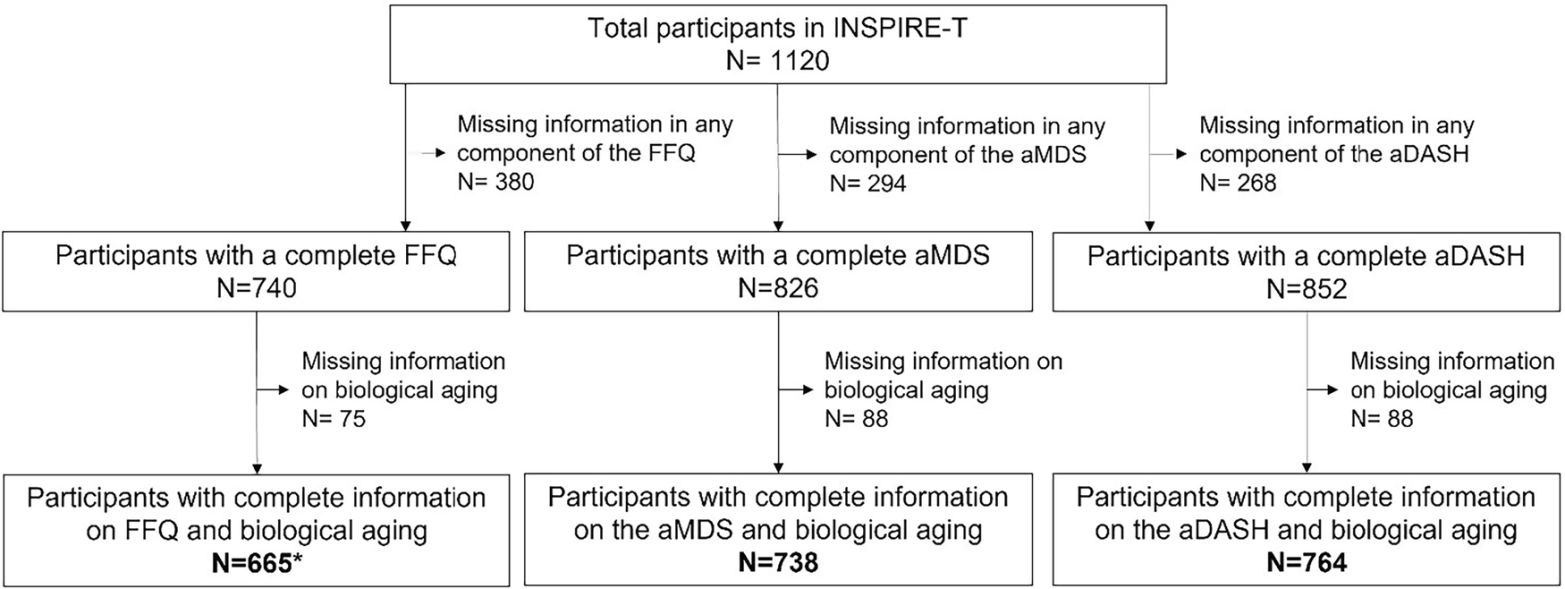
Flowchart of the study. *One participant was excluded after visual inspection (scatterplot and boxplot) of biological age acceleration, resulting in a sample of 664^51^.

We performed cross-sectional analyses of baseline measures from participants in the INSPIRE Human Translational Cohort (INSPIRE-T cohort) (clinical database version 2025.1), included between October 2019 and December 2024. INSPIRE-T cohort is an ongoing 10-year observational study involving adults aged 20 years and over (with no upper age limit) in Southwestern France^21^. Briefly, to be included, individuals must be 20 years or older, be affiliated to a social security scheme, not have a severe disease that compromises life expectancy over the next 5 years (or 1 year, in the case of individuals dependent on daily life activities), and not be deprived of their liberty by administrative or judicial decision, or under guardianship. In our study, we included only those with complete data on dietary intake and biological age clocks (Fig. 1). The INSPIRE-T protocol was approved by the French Ethics Committee in Rennes (CPP Ouest V) in October 2019, and it is available at “clinicaltrials.gov” (ID NCT04224038; registration date: October 15, 2019). The French National Commission for Data Protection gave its authorization in April 2017 (Ref. Nb. MMS/OSS/NDT171027). All participants provided their written consent, and the study was conducted following the 1964 Declaration of Helsinki. No further IRB approval was necessary for the present study after verification by the INSPIRE-T data access committee.

### Dietary intake: exposure factor

Dietary intake, referred to the previous four months, was assessed through a qualitative 34-item Food Frequency Questionnaire (FFQ) based on the study of Estaquio et al.^22^. The frequencies of consumption (never or rarely; less than once a week; once a week; two or three times a week; four to six times a week; once or more a day) were converted to a daily frequency by dividing the weekly frequency by 7 days (0; 0.071; 0.143; 0.358; 0.714; 1 or more times per day).

### Dietary patterns a posteriori

The derivation of the DPs followed the same strategies applied in a previous study within the same cohort^18^. First, the food items were reduced into 18 food groups (Supplementary Table 1), followed by a data-driven principal component analysis (PCA). The PCA generates DPs (factors) by aggregating the food groups according to their intercorrelation. Three main DPs were retained based on the eigenvalue greater than 1.30^23^ and the examination of the breaking point in Cattell’s scree test^24^. Data adequacy was confirmed by having a significant Bartlett test of sphericity (*p* < 0.001) and a Kaiser–Meier–Olkin (KMO) test equal to 0.589 (0.5 is the minimal acceptable)^24^. The three DPs accounted for 28.8% of the total variance, which is in line with studies in nutritional epidemiology^24,25^.

The factors were rotated by the orthogonal transformation VARIMAX to maximize the differences and facilitate interpretation. Food groups were kept when they explained at least 10% of the variance of one DP^24^. We considered only food groups with rotated factor loadings ≥0.30 for interpreting and labeling the DPs, as these variables contribute meaningfully (i.e., show higher correlation with the corresponding factor)^24^. Three DPs were extracted: (1)“Plant-based”, with positive factor loadings for breakfast cereals, legumes, wholegrains, fruits and vegetables, and oil; (2) “Sugar and fast food”, in which there were positive loadings for salted biscuits, industrialized meals, soft drinks, desserts, sweets and pastries, and (3) “Sandwiches”, consisting of positive factor loadings for charcuterie (lunch meats and offal), refined starch (e.g., white bread), butter or margarine, and oil (Supplementary Tables 1 and 2).

Participant-specific DP scores were calculated as weighted linear combinations of standardized food group intakes using the scoring coefficients from the varimax-rotated solution. These scores reflect the degree of conformity to each identified pattern, with higher values indicating greater conformity to the corresponding DP.

### Dietary patterns a priori

Adherence to a priori DPs was assessed using adapted scoring systems for the Mediterranean diet and the DASH.

Mediterranean diet adherence was assessed through an adapted version of the Mediterranean Diet Scale (aMDS): participants above the sex-specific median intake of vegetables (excluding potatoes), fruits, wholegrains, legumes, and fish received 1 point, and those below the median were scored as 0. As our FFQ was qualitative, which does not allow for calculating nutrients, we considered the amount of oil intake instead of the ratio of monounsaturated to saturated lipids^26^; then, we assigned the same scoring method (1 when above and 0 when below the median). The inverse scores were attributed to meat (red and processed meat, and poultry) and fatty dairy intake—those below the median received 1 point (Supplementary Table 3). Regarding alcohol consumption, this information was obtained as “glass/day” and then converted to ethanol/day, considering that 1 glass has a 12 g ethanol concentration^27^. We assigned 1 point when alcohol intake was between 10 and 50 g/day for males and between 5 and 25 g/day for females^28^. As we did not have this information for the entire population, we decided to exclude alcohol from the total aMDS. The possible score range for aMDS was then 0 (minimal adherence) to 8 (maximal adherence). We performed an exploratory analysis with the subsample with a complete aMDS, in which alcohol intake was included (0–9) (Supplementary Tables 10–12).

DASH—to check the adherence to this diet, we adapted the calculation from the DASH score proposed by Fung et al.^29^. We constructed the DASH score based on 7 diet components: fruits, vegetables, nuts and legumes, low-fat dairy products, whole grains (beneficial components), sweetened beverages, and red and processed meats (detrimental components) (Supplementary Table 3). Because sodium was not measurable within our FFQ, we did not include it in the score computation. The daily intake of the seven food groups was divided into tertiles, with the beneficial components receiving 1 point when intake was in the first tertile, 2 points when in the second tertile, and 3 points when in the third tertile. Inverted scores were attributed to the detrimental components. The overall adapted DASH score (aDASH) was calculated by summing the component scores, with possible results ranging from 7 to 21—the highest indicating the greatest adherence.

No additional confirmation procedures were performed for either a priori or PCA-derived DPs, as scores were directly computed from the FFQ data using standard methods.

### Biological age acceleration: the outcome

#### Epigenetic clocks

Genomic DNA was extracted from frozen blood samples (collected at baseline) from the INSPIRE-T cohort using the Qiagen DNeasy Blood & Tissue kit (Qiagen N.V., Venlo, Netherlands). Following bisulfite conversion, DNA methylation was profiled using the Illumina Infinium MethylationEPIC BeadChip (EPIC v1; Illumina Inc., San Diego, USA) according to the manufacturer’s instructions. Raw intensity data were processed using Partek® Genomics Suite® (Partek Inc., Chesterfield, USA) to obtain methylation β-values ranging from 0 (completely unmethylated) to 1 (completely methylated). No additional normalization was applied within Partek before epigenetic clock estimation. Horvath’s clock (353 CpGs)^30^, Hannum’s clock (71 CpGs)^4^, and PhenoAge clock (Levine; 513 CpGs)^31^ (Levine; 513 CpGs) were calculated using the methylclock R package. Normalization was enabled (normalize = TRUE), applying the BMIQ-based normalization procedure implemented in the package. When required, missing CpGs were handled using the default internal imputation procedure (fastImp = FALSE). GrimAge (1030 CpGs) was calculated following the original method described by Lu et al.^7^. Normalization was performed in accordance with the recommended settings (BMIQ-based normalization), and k-nearest neighbor (kNN) imputation was applied to handle missing CpGs when necessary.

Epigenetic age acceleration was defined as the residuals obtained from linear regression models of each DNAm age estimate on chronological age. For Horvath’s, Hannum’s, and PhenoAge clocks, residuals were further adjusted for estimated blood cell counts (neutrophils, basophils, monocytes, lymphocytes, and eosinophils). Positive residuals indicate accelerated biological aging.

#### Inflammatory aging clock

The iAge was estimated from the baseline blood sample following the procedures outlined in Sayed et al.^8^. Serum samples were diluted threefold in Luminex assay buffer and analyzed using a Luminex L200 with a custom ProCartaPlex Luminex kit (Thermo Fisher, Santa Clara, USA). For quality control, assay chex beads were added to each well (Radix Biosolutions, Georgetown, USA). Samples were run in duplicate and incubated overnight at 4 °C with the Luminex beads. The trimmed mean intensity values were averaged for each sample. The trimmed distribution includes the events collected for each test in a single sample, with the lowest 5% and highest 5% of data points removed. Analyte levels were transformed to match the scale of the Stanford 1KIP data using protein standards provided with the Luminex kit on each plate, and these standards were also run on a bridge plate containing samples that had been processed on a bridge plate with samples from 1KIP. All analyses were conducted using the average trimmed mean intensity values.

A linear regression model was trained using the five circulating inflammatory analytes—CCL11 (eotaxin-1), CXCL1 (GRO-α), CXCL9, IFN-γ, and the TNF-related apoptosis-inducing ligand (TRAIL)—as predictor variables. These were identified in the INSPIRE-T cohort as the strongest contributors to iAge and were used to compute the iAge score. In the original report describing iAge, Sayed et al. found no significant difference in prediction error between this five-analyte model and a model incorporating 50 inflammatory markers, supporting the use of the reduced feature set without loss of accuracy.

The inflammatory age acceleration was estimated from the residuals of the regression of chronological age and iAge, with positive values indicating that biological age exceeds chronological age.

### Confounders

Through baseline questionnaires we assessed: chronological age (20–44, 45–64, ≥65)^32^, sex (male, female), number of medications, medical history of chronic diseases, educational level (no schooling/primary, middle school/high school, college degree or above), and income (<1500€, 1500€ to 2800€, 2800 to 4200€, >4200€, don’t know/don’t want to respond). Physical activity during leisure time and transport (MET-min/week) was obtained using the long version of the International Physical Activity Questionnaire (IPAQ)^33^. The Body Mass Index (BMI; kg/m^2^) was obtained by dividing measured weight (kg) by height squared (m^2^).

### Mediators

Body composition was assessed by Dual Energy X-Ray absorptiometry (DXA; GE Healthcare Lunar iDXA). We tested the mediating role of total body fat (as a percentage of total body mass - %), android fat (i.e., abdominal region, as a percentage of total body mass - %), and android-to-gynoid fat ratio (i.e., the ratio of the fat around the trunk/abdominal area to the fat around the femoral-hip area) on the relationship between DPs and biological age acceleration.

### Statistics and reproducibility

Descriptive analyses were performed, with the results being presented as median (25th–75th percentile) and absolute frequency (%). Descriptive data were compared between males and females by using the Mann-Whitney test for continuous variables and the chi-squared test for categorical variables.

We performed linear regression models between each DP and each biological age acceleration indicator (treated as continuous). Because the data-driven (*a posteriori*) DP scores were negatively skewed and included negative values, natural log transformation was applied to improve model fit and satisfy linear regression assumptions. For each DP, a constant equal to the absolute value of the minimum observed score plus 1 was added to all observations before transformation to ensure positive values (|minimum DP score| + 1). The constants were 3.75 for the “Plant-based” DP, 4.10 for the “Sugar and fast-food” DP, and 4.99 for the “Sandwiches” DP. The transformed scores were entered as continuous variables in the regression analyses. The scores for aMDS and aDASH were treated as ordinal variables. After running a crude model (Model 1), we included categories of age, sex, number of medications, medical history of diseases, educational level, income, and physical activity (Model 2). Model 3 was further adjusted for BMI. The assumptions of linearity, normality, and homoscedasticity were verified graphically through scatterplots, histograms, quantile–quantile plots, and residual plots (Supplementary Figs. 1–15). We examined the moderating effect of sex and age categories by including interaction terms in Model 3. Cook’s distance (measures overall influence) was plotted with leverage (hat values) to check for influential observations^34^.

The mediating roles of total body fat (%), android fat (%), and the android-to-gynoid fat ratio were examined using Structural Equation Modeling (SEM), while adjusting for the same confounders as in Model 2. The indirect association for each model was bootstrapped (1000 resamples) to avoid bias on data normality and to provide more robust confidence intervals. No adjustments were made for multiple comparisons, as analyses were based on prespecified hypotheses.

All analyses were performed using the available data by using Stata v19 (StataCorp LLC, College Station, Texas, USA). Two-sided *p*-values were reported, with a statistical significance set at *α* = 0.05.

## Results

From the 1120 participants in the INSPIRE-T cohort, we include 764 individuals (62% female), 20–100 years (42% older than 65), with complete information on diet and biological clocks (Fig. 1). This is an apparently healthy population (69% take a maximum of 2 medications and 48% have no medical history of any specific chronic disease), with a high level of education (75%) and belonging to the middle-to-upper socioeconomic class (58%) (Table 1).

**Table 1:** Descriptive characteristics of the included population. The values are presented as median (25th–75th) or frequency (%)

Variables	Total (*n* = 764)	Male (*n* = 289; 37.8%)	Female (*n* = 475; 62.2%)	*p*
Chronological age (years)	61 (45; 73)	64 (49; 75)	58 (44; 72)	0.002
Age group				0.005
Younger (≥20 and <45)	181 (23.7)	58 (32.0)	123 (68.0)	
Middle age (≥45 and <65)	259 (33.9)	87 (33.6)	172 (66.4)	
Older (≥65)	324 (42.4)	144 (44.4)	180 (55.6)	
Horvath’s clock (years)	59.62 (47.75; 69.58)	63.80 (53.21; 73.49)	57.22 (45.64; 67.22)	0.000
Hannum’s clock (years)	49.66 (38.22; 60.46)	54.92 (41.57; 63.79)	47.23 (36.56; 58.44)	0.000
PhenoAge (years)	45.22 (32.16; 56.73)	49.57 (35.78; 61.16)	42.90 (30.12; 54.67)	0.000
GrimAge (years)	60.40 (48.91; 70.87)	65.44 (51.58; 75.39)	57.69 (47.30; 67.08)	0.000
iAge (years)	56.02 (50.43; 64.14)	56.68 (51.13; 64.53)	55.84 (50.22; 63.67)	0.202
Plant-based DP (*n* = 664)	−0.17 (−1.06; 0.83)	−0.37 (−1.23; 0.46)	−0.02 (−0.97; 0.97)	0.002
Sugar and fast-food DP (*n* = 664)	−0.27 (−0.86; 0.57)	−0.01 (−0.71; 0.89)	−0.37 (−0.92; 0.40)	0.000
Sandwiches DP (*n* = 664)	−0.09 (−0.86; 0.73)	−0.03 (−0.89; 1.02)	−0.15 (−0.85; 0.60)	0.114
aMDS (*n* = 738)	4 (3; 5)	4 (4; 5)	4 (3; 5)	0.219
aDASH (*n* = 764)	13 (11; 15)	12 (11; 14)	13 (11; 15)	0.000
FFQ application: season of the year (*n* = 764)				0.127
Winter	218 (28.5)	95 (32.9)	123 (25.9)	
Spring	147 (19.2)	58 (20.1)	89 (18.7)	
Summer	204 (26.7)	68 (23.5)	136 (28.6)	
Autumn	195 (25.5)	68 (23.5)	127 (26.7)	
BMI (kg/m^2^)	24.44 (21.83; 27.27)	25.66 (23.28; 28.24)	23.58 (21.28; 26.50)	0.000
Total body fat (%)	31.84 (25.84; 37.80)	27.39 (21.62; 31.92)	35.56 (29.42; 40.45)	0.000
Android fat (%)	2.59 (1.72; 3.41)	2.67 (1.77; 3.49)	2.54 (1.66; 3.40)	0.194
Android/Gynoid ratio	0.46 (0.33; 0.65)	0.68 (0.50; 0.83)	0.38 (0.28; 0.48)	0.000
Physical activity (MET-min/week)^a^	1076 (448; 2147)	1298 (495; 2400)	954 (393; 1908)	0.005
Income (*n* = 755)				0.004
<1500€	75 (9.9)	21 (28)	54 (72)	
1500€ à 2800€	199 (26.4)	62 (31.2)	137 (68.8)	
2800 à 4200€	227 (30.1)	84 (37)	143 (63)	
≥4200€	217 (28.7)	103 (47.5)	114 (52.5)	
Don’t know/respond	37 (4.9)	15 (40.5)	22 (59.5)	
Marital status (*n* = 760)				0.000
Married/couple	498 (65.5)	221 (44.4)	277 (55.6)	
Single/divorced/widower	262 (34.5)	67 (25.6)	195 (74.4)	
Education level (*n* = 762)				0.287
No schooling/primary	29 (3.8)	15 (51.7)	14 (48.3)	
Middle/high school	157 (20.6)	58 (36.9)	99 (63.1)	
College or higher	576 (75.6)	215 (37.3)	361 (62.7)	
Medications taken (*n* = 764)				0.905
0	272 (35.6)	107 (39.3)	165 (60.7)	
1–2	256 (33.5)	93 (36.3)	163 (63.7)	
3–4	111 (14.5)	41 (36.9)	70 (63.1)	
5 or more	125 (16.4)	48 (38.4)	77 (61.6)	
Medical history of chronic diseases (*n* = 754)				0.000
0	365 (48.4)	118 (32.3)	247 (67.7)	
1–2	271 (35.9)	105 (38.8)	166 (61.3)	
3 or more	118 (15.7)	62 (52.5)	56 (47.5)	

*DP* dietary pattern (two-sided *p*-values)

^a^Physical activity in leisure time and transport. Mann-Whitney and chi-squared.

Females were younger than males according to their chronological and epigenetic ages, had lower BMI but higher body fat (predominantly in the lower body), and spent less time in physical activities during their leisure time and while commuting. These results were consistent in sensitive analysis with a smaller sample with complete information for DPs *a posteriori* (*n* = 664; Supplementary Table 4). Regarding the dietary intake, females presented higher scores for the “Plant-based” DP and the aDASH, indicating greater conformity to these DPs, and lower scores for the “Sugar and fast-food” DP.

### Associations between DPs and biological age acceleration

The linear regression models adjusted for most confounders (Model 2) showed that higher adherence to aDASH was significantly associated with lower PhenoAge acceleration (*β* = −0.17, 95% CI: −0.31; −0.02). Similarly, the higher the scores for a “Plant-based” diet, the lower the PhenoAge acceleration, although this association was only marginally significant (*β* = −1.09, 95% CI: −2.19; 0.01). Both aDASH and “Plant-based” DP lost significance when the inclusion of BMI in the model (Model 3), which suggests a role of body composition. No significant associations were observed for the other DPs, first-generation epigenetic clocks, GrimAge, or iAge (Table 2).

**Table 2:** Linear regression models

Dietary pattern	Model 1 β (95% CI)	p*	Model 2 β (95%CI)	p*	Model 3 β (95% CI)	p*
Biological age acceleration by Horvath’s clock						
a posteriori DPs	*N* = 664		*N* = 650		*N* = 610	
Plant-based	0.08 (−0.99; 0.84)	0.869	0.34 (−0.61; 1.30)	0.481	0.46 (−0.53; 1.45)	0.363
Sugar and Fast food	0.21 (−0.93; 1.36)	0.713	0.26 (−0.96; 1.48)	0.675	0.39 (−0.86; 1.64)	0.542
Sandwiches	0.36 (−0.90; 1.63)	0.572	0.20 (−1.11; 1.52)	0.760	0.15 (−1.20 −1.51)	0.825
a priori DPs	(aMDS *n* = 738 / aDASH *n* = 764)		(aMDS *n* = 673 / aDASH *n* = 686)		(aMDS *n* = 621 / aDASH *n* = 629)	
aMDS	−0.05 (−0.28; 0.17)	0.642	−0.04 (−0.26; 0.19)	0.754	0.03 (−0.21; 0.26)	0.817
aDASH	−0.13 (−0.25; 0.00)	0.054	−0.05 (−0.18; 0.08)	0.443	−0.02 (−0.15; 0.12)	0.824
Biological age acceleration by Hannum’s clock						
a posteriori DPs						
Plant-based	−0.08 (−0.78; 0.62)	0.827	0.18 (−0.57; 0.92)	0.686	0.05 (−0.74; 0.84)	0.902
Sugar and Fast food	−0.26 (−1.14; 0.61)	0.555	−0.52 (−1.48; 0.42)	0.278	−0.41 (−1.41; 0.58)	0.417
Sandwiches	−0.08 (−1.05; 0.89)	0.870	−0.31 (−1.34; 0.72)	0.558	−0.20 (−1.28; 0.88)	0.716
a priori DPs						
aMDS	−0.07 (−0.24; 0.10)	0.439	−0.06 (−0.23; 0.11)	0.488	−0.09 (−0.27; 0.10)	0.363
aDASH score	−0.07 (−0.16; 0.03)	0.169	−0.02 (−0.12; 0.08)	0.699	−0.04 (−0.14; 0.07)	0.495
Biological age acceleration by PhenoAge						
a posteriori DPs						
Plant-based	−1.20 (−2.24; −0.16)	0.024	−1.09 (−2.19; 0.01)	0.051	−0.87 (−2.00; 0.27)	0.135
Sugar and Fast food	0.68 (−0.62; 1.98)	0.303	1.07 (−0.33; 2.47)	0.135	0.88 (−0.55; 2.32)	0.226
Sandwiches	−0.64 (−2.09; 0.80)	0.382	−0.75 (−2.26; 0.77)	0.334	−0.92 (−2.48; 0.64)	0.248
a priori DPs						
aMDS	−0.25 (−0.51; 0.00)	0.052	−0.23 (−0.48; 0.03)	0.082	−0.16 (−0.42; 0.11)	0.252
aDASH	−0.20 (−0.34; −0.05)	0.007	−0.17 (−0.31; −0.02)	0.028	−0.15 (−0.30; 0.01)	0.066
Biological age acceleration by GrimAge						
a posteriori DPs						
Plant-based	−0.56 (−1.28; 0.12)	0.107	0.11 (−0.60; 0.81)	0.762	0.27 (−0.44; 0.99)	0.450
Sugar and Fast food	0.98 (0.10; 1.86)	0.029	−0.25 (−1.15; 0.65)	0.589	−0.45 (−1.36; 0.45)	0.328
Sandwiches	0.73 (−0.25; 1.70)	0.144	−0.04 (−1.02; 0.93)	0.931	−0.18 (−1.16; 0.80)	0.719
a priori DPs						
aMDS	0.05 (−0.11; 0.22)	0.530	0.07 (−0.09; 0.23)	0.389	0.12 (−0.05; 0.28)	0.174
aDASH	−0.16 (−0.24; −0.07)	0.001	−0.03 (−0.13; 0.06)	0.470	−0.04 (−0.13; 0.06)	0.426
Biological age acceleration by iAge						
a posteriori DPs						
Plant-based	0.15 (−1.55; 1.85)	0.867	0.29 (−1.54; 2.13)	0.753	0.02 (−1.90; 1.95)	0.981
Sugar and Fast food	−0.06 (−2.20; 2.07)	0.954	0.21 (−2.13; 2.57)	0.858	0.19 (−2.25; 2.63)	0.879
Sandwiches	−1.59 (−3.94; 0.76)	0.184	−0.77 (−3.29; 1.75)	0.546	−0.75 (−3.37; 1.88)	0.576
a priori DPs						
aMDS	0.11 (−0.29; 0.51)	0.596	0.15 (−0.28; 0.58)	0.492	0.16 (−0.30; 0.61)	0.499
aDASH	−0.00 (−0.23; 0.22)	0.978	−0.01 (−0.26; 0.23)	0.908	−0.03 (−0.29; 0.23)	0.838

A posteriori DPs were log transformed.

Model 1: unadjusted.

Model 2: age categories, sex, total medications, current diseases, educational level, income range, and physical activity in leisure time and transport.

Model 3: Model 2 + BMI.

*Two-sided t-test.

### Moderating effect of sex and chronological age

**Fig. 2:**
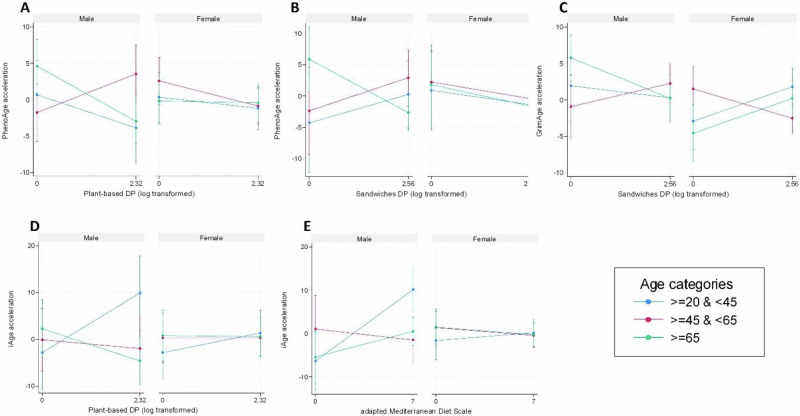
Significant interactions with age category and sex. Linear regression models adjusted for categories of age, sex, number of medications, medical history of diseases, educational level, income, physical activity, and BMI. Error bars represent 95% confidence intervals. No adjustments were made for multiple comparisons. **A** (*n* = 563): The slope for older men (≥65) was significant (*p* = 0.021) and significantly differed from the middle-aged men (*p* = 0.011) (two-sided t-test). **B** (*n* = 563): The slope for older men (≥65) was significant (*p* = 0.033) and significantly differed from middle-aged men (*p* = 0.049) (two-sided t-test). **C** (*n* = 561): The slope for older men (≥65) was significant (p = 0.026) and significantly differed from middle-aged men (*p* = 0.044), younger women (*p* = 0.012), and older women (*p* = 0.010) (two-sided t-test). **D** (*n* = 556): The slope for younger men (≥20–<45) was significant (*p* = 0.032) and significantly different from older men (*p* = 0.013) (two-sided t-test). **E** (*n* = 613): The slope for younger men (≥20–45) was significant (*p* = 0.003) and significantly differed from young women (*p* = 0.025), middle-aged men (*p* = 0.024), middle-aged women (*p* = 0.004), and older women (*p* = 0.005) (two-sided t-test)

We observed a significant three-way interaction between diet, sex, and age category in relation to the “Plant-based” and “Sandwiches” DPs and epigenetic age acceleration. Specifically, higher scores on the “Plant-based” and “Sandwiches” DPs were associated with lower PhenoAge acceleration in older males only (Fig. 2 and Supplementary Table 5). A similar inverse association in older males with the “Sandwiches” DP was found using GrimAge. An unexpected result arose from iAge in young males, in whom higher scores on the “Plant-based” DP and aMDS were associated with markedly higher iAge acceleration.

Exploratory additional two-way interactions (i.e., diet x sex and diet x age) did not result in further significant moderating effects (Supplementary Table 6).

### Mediating effect of body fat

**Fig. 3:**
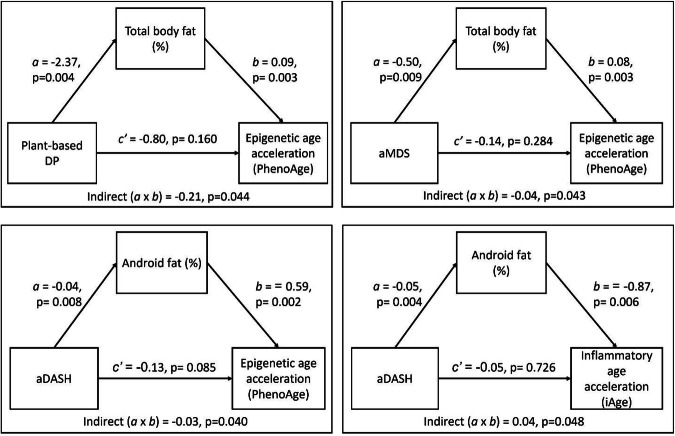
Frameworks of the structured equation models (SEM) for the significant associations of dietary patterns and biological age acceleration. All models were adjusted for age categories, sex, education, income, medical history of diseases, medication, and physical activity (two-sided z-test). No adjustments were made for multiple comparisons.

Body fat partially explained the association between certain DPs and biological age acceleration (Fig. 3 and Supplementary Tables 7–9): higher scores on the “Plant-based” DP, aMDS, and aDASH were associated with lower PhenoAge acceleration, and these associations were statistically explained, in part, by differences in body fat. Specifically, higher scores on these DPs were associated with lower levels of total or android fat, which in turn were associated with reduced PhenoAge acceleration. The inclusion of total body fat explained 26% of the observed association between the “Plant-based” DP and epigenetic age acceleration, and 29% for aMDS, while the inclusion of android fat accounted for 23% of the association between aDASH and PhenoAge acceleration.

Another unexpected result arose for iAge, with higher adherence to aDASH being indirectly associated with higher iAge acceleration, which was partly explained by differences in android fat (indirect association: *β* = 0.04, *p* = 0.048).

### Additional exploratory analyses

In the exploratory analyses within the subsample with complete information on the aMDS (*n* = 238)—inclusion of alcohol as a beneficial component– the higher the adherence to this DP, the higher the GrimAge acceleration (Model 3: *β* = 0.30, 95% CI: 0.05; 0.55) (Supplementary Table 10). This association was particularly true among middle-aged females (*p* = 0.049) (Supplementary Table 11) and was not explained by body fat (Supplementary Table 12). On the contrary, middle-aged and older females were suggested to have lower iAge acceleration when adhering to this complete MDS (Supplementary Table 11).

## Discussion

We observed that diets predominantly composed of wholegrains, legumes, fruits, vegetables, and low-fat dairy were associated with lower biological aging, and these associations were partially explained by reductions in body fat. Specifically, a 10-point increase in aDASH score was associated with a 1.7-year lower PhenoAge, with android fat accounting for 23% of this association. Further, every one-unit increase in scores for the “Plant-based” DP was associated with a 1.1-year lower PhenoAge, with 26% of this association being explained by the total body fat.

It has been previously demonstrated that higher adherence to the DASH diet was associated with lower PhenoAge acceleration^35^. Indeed, it is suggested an inverse relationship between common food components of the “Plant-based” DP and aDASH and biological aging, with the higher the intake of grains, vegetables, and fruits, the lower the epigenetic age acceleration^36,37^. The analysis of approximately 79,000 participants in the UK Biobank showed that substituting 5% energy from animal protein for 5% energy from plant protein was associated with 12–28% lower odds of biological aging, especially when substituting red meat for wholegrains or nuts^38^. Interestingly, replacing yogurt with legumes went in the opposite direction, which may explain our more consistent results coming from aDASH, which includes low-fat dairy as a beneficial component (Supplementary Table 3).

Dwaraka et al. found a reduction in epigenetic age acceleration after an 8-week vegan diet (avoiding all animal products) among 20 pairs of twins (mean age 40 years). The same was not observed when an omnivorous diet was followed. One possible explanation is that the vegan cohort had lower caloric intake than the omnivorous group^39^. Caloric restriction is suggested to promote a drift in DNA methylation in animal models^40^ and –in less extend—humans^41^. Overweight young women following a diet with 30% fewer calories (∆600 kcal) had reductions in DNA methylation in the *CD36* gene in blood cells (in CpG +477), which has a role in human lipid metabolism^41^.

The lower energy intake typically observed with plant-based diets may explain the role of body fat in our mediation model. Clinical indicators of obesity, such as the waist-hip ratio and BMI, as well as more direct indicators, such as visceral and subcutaneous abdominal fat, have been positively associated with epigenetic age acceleration^42–44^. A full two-way Mendelian randomization study using European databases showed a significant, causal, and robust association between BMI and epigenetic aging^45^. Taken together, the current evidence leads us to hypothesize that a healthy plant-based diet may result in lower caloric intake, leading to a reduction in body fat, which in turn slows epigenetic aging.

We observed some moderating effects of age and sex, with the significant association between the “Plant-based” DP and lower PhenoAge acceleration happening only in older males. Because males start with faster biological aging^16,46^—as also evidenced in our population—they may derive stronger epigenetic responses from bioactive plant compounds. Meanwhile, females showed higher scores for plant-based diets, so the relative association may appear more modest.

Some unexpected results emerged from iAge. Among younger males (20–44 years), greater iAge acceleration was observed with higher scores for the “Plant-based” DP and the aMDS. Additionally, mediation analysis revealed a positive indirect association between aDASH and higher iAge acceleration. The mechanisms underlying these associations remain unclear; therefore, the following hypotheses are intended as conceptual explanations and should be interpreted cautiously until validated in mechanistic or longitudinal studies. First, caloric restriction in non-obese younger adults may lead to reductions in body fat that could increase cortisol levels, potentially modulating immune function^47^. Elevated cortisol has been associated with immune suppression and alterations in immune cell distribution and behavior, which could reduce immune surveillance and increase susceptibility to inflammatory conditions^48^. Second, the theory of antagonistic pleiotropy suggests that genetic or biological processes advantageous earlier in life may exert deleterious effects later^49^. Immune or metabolic adaptations beneficial in younger individuals could contribute to pro-inflammatory signatures reflected in iAge. It is important to consider that the subgroup of young males was composed of a relatively small number of people (*n* = 55) and that unmeasured factors known to affect inflammatory pathways, such as psychosocial stress and risky behaviors (e.g., smoking), may be influencing this association.

Exploratory analysis with the subsample having complete MDS (adding alcohol intake) showed that higher adherence to this diet was directly associated with higher GrimAge acceleration. Seeking factors causally linked to epigenetic age acceleration, Kong et al. found that for GrimAge, higher alcohol intake was the second strongest risk factor^50^, which may explain the controversial results after including alcohol as a beneficial component of the MDS composite.

We did not find significant associations when using first-generation epigenetic clocks. Horvath’s and Hannum’s clocks were developed to predict chronological age, whereas PhenoAge and GrimAge were trained to estimate biological age and mortality risk using a composite of biomarkers^3^. Harris et al.^51^ found that social and lifestyle factors were associated with biological aging as measured by second- and third-generation clocks. These associations suggest that the newer clocks are sensitive indicators of biological aging processes before the onset of age-related disease comorbidities, serving as useful surrogate endpoints in interventions targeting determinants of healthy aging.

Our findings should be interpreted with caution due to study limitations. First, the cross-sectional design precludes establishing causality or directionality of associations, and it does not account for seasonal or life-course variations or long-term changes in biological markers. The use of a qualitative FFQ limits exploring the role of caloric intake and assessing nutrient levels. Furthermore, the lack of lifetime dietary information beyond the previous 4 months limits the ability to capture historical variations in DPs. Nevertheless, longer recall periods in self-reported dietary assessment may increase memory-related measurement error and misclassification. The lack of complete data on alcohol and smoking, along with other potential unmeasured confounders, may have influenced the results. The specific characteristics of our study population—including sample size, European background, high socioeconomic status, and generally good health—also limit the generalizability of our findings. Nonetheless, we employed multiple statistical approaches, including moderation and mediation analyses, to address potential data limitations. We also examined the influence of body fat using DXA, a gold-standard method. Lastly, our analyses included both data-driven DPs and well-established recommended diets, within a sample covering a wide age range.

## Conclusion

Greater scores in the “Plant-based” DP and DASH diet, mainly characterized by higher intakes of wholegrains, legumes, fruits, vegetables, and low-fat dairy, were associated with lower epigenetic age acceleration, partially explained by reductions in body fat. These findings need to be further explored in longitudinal studies and clinical trials.

## Supplementary information

Transparent Peer Review file

Supplemental material

## Data Availability

The data supporting the findings of this study are available upon reasonable request, following approval of a methodologically sound research proposal by the INSPIRE-T data access committee and the signature of a data use agreement. The utilization of the data supplied other than as described by the recipient in the research proposal is strictly prohibited. In case of substantial modification to the initial study proposal, including the need for an extension on the project’s duration, the recipient must submit an updated Data Access Application. Requests are reviewed in an ongoing fashion at monthly data access committee meetings, and requestors are informed of the committee’s decision following each meeting. Inquiries or proposals should be addressed to https://ihuos_inspiredataaccess@chu-toulouse.fr (CC: nicola.coley@utoulouse.fr).
