# Supplementary material for: The role of dietary patterns on epigenetic and inflammatory aging based on the INSPIRE-T study
Source: Commun Med (Lond). 2026 May 13;6:410. doi: 10.1038/s43856-026-01643-1 (PMC13396666; doi:10.1038/s43856-026-01643-1)
Supplement: Supplementary file 2 — Supplemental material [file 43856_2026_1643_MOESM2_ESM.pdf]

SUPPLEMENTARY INFORMATION

Supplementary tables

Supplementary table 1 Description of the food items from the FFQ in each food group

Food groups	Foods in each group
Meat and poultry	Beef, veal, lamb, pork, chicken, turkey
Fish	Fish and seafood
Eggs	Eggs
Charcuterie	Lunch meat and offal (ham, pâté, bacon, sausage, andouillette)
Breakfast cereals	Breakfast cereals
Legumes	lentils, kidney beans, chickpeas, etc.
Salted biscuits	Salted snacks and crackers, peanuts, and other appetizers
Dairy products	Milk, Swiss cheese, yoghurt, cottage cheese, cheeses, including low-fat dairy products
Starch	White bread, rusks, pasta, potatoes, rice, semolina, etc.
Wholegrain starch	Wholegrain, brown, buckwheat, cereal, rye bread, or crackers<br>Wholegrain or brown rice, wholegrain pasta
Fruits and vegetables	Raw or cooked vegetables<br>Fresh fruit and fresh juice
Commercial meals	Pre-prepared/prepared meals (canned, frozen, delicatessen: couscous, cassoulet, sauerkraut, etc.), low-fat pre-prepared meals, fast foods (hamburgers, kebab, sandwiches, pizza, quiches), fried food (French fries, doughnuts, breaded meat or fish)
Butter and margarine	Butter and margarine for breakfast, as a side dish, in meal preparation
Oil	Oil
No sugar beverages	Coffee and tea
Soft drinks	Light soda, soda, and energy drinks
Industrialized juice	Juice or nectar
Desserts, sweets, and pastries	Sweet desserts (entremets, cream desserts, mousses, ice creams), pastries, cakes, sweet cookies, chocolate or cereal bars, candies, chocolate, etc.

Supplementary table 2 Correlation coefficient of each food group with the three dietary patterns

Food groups	Plant-based (variance: 10%)	Sugar and fast food (variance: 9.3%)	Sandwiches (variance: 9.5%)
Meat and poultry	-0.18	0.04	0.29
Fish	0.02	-0.22	0.05
Eggs	0.17	-0.00	0.03
Charcuterie	-0.05	0.22	0.36*
Breakfast cereals	**0.32***	0.19	-0.27
Legumes (lentils, beans, etc)	**0.39***	0.07	-0.21
Salted biscuits	-0.06	0.32*	0.10
Dairy products	0.26	0.16	0.22
Starch	-0.02	0.05	0.50*
Wholegrain starch	0.41*	-0.02	-0.11
Fruits and vegetables	0.49*	-0.06	0.15
Commercial meals	-0.01	0.55*	-0.07
Butter and margarine	0.07	-0.14	0.44*
Oil	0.34*	-0.13	0.34*
No sugar beverages	0.27	0.01	0.06
Soft drinks	-0.00	0.40*	0.01
Commercial juice	-0.02	0.23	0.02
Desserts, sweets, and pastries	0.04	0.41*	0.06

*Factor loadings ≥0.30 are considered for labeling the patterns

Supplementary table 3 Food groups of the adapted Mediterranean Diet scale (aMDS) and the adapted Dietary Approach to Stop Hypertension (aDASH)

aMDS	aDASH
Beneficial components	Beneficial components
• Legumes: legumes, beans, peas, etc<br>• Fruits: fresh fruits and fresh juices<br>• Vegetables (raw or cooked)<br>• Fish and seafood<br>• Wholegrain starch: wholegrain, brown, buckwheat, cereal, rye bread, or crackers, wholegrain or brown rice, wholegrain pasta<br>• Oil	• Legumes: legumes, beans, peas, etc<br>• Fruits: fresh fruits and fresh juices<br>• Vegetables (raw or cooked)<br>• Wholegrain starch: wholegrain, brown, buckwheat, cereal, rye bread, or crackers, wholegrain or brown rice, wholegrain pasta<br>• Low-fat dairy: low-fat milk, cheese, and yogurt
Detrimental components	Detrimental components
• Meat and poultry: beef, veal, lamb, pork, poultry, lunch meat, and offal<br>• Full-fat dairy: fatty milk, cheese, and yogurt	• Red fresh and processed meat: beef, veal, lamb, pork, lunch meat, and offal<br>• Sweetened beverages: soft drinks, energy drinks, industrialized juices

Supplementary table 4 Descriptive characteristics of the smaller sample (n=664) of the included population (for a posteriori DP). The values are presented as median (25th; 75th) or frequency (%)

Variables	Total (n= 664)	Male (n= 246; 37.1%)	Female (n= 418; 62.9%)	p-value
Chronological age (years)	59.5 (44; 73)	64 (47; 74)	56 (41; 72)	0.004
Age group				0.030
Younger (>=20 & <45)	173 (15.57)	53 (30.64)	120 (69.36)	
Middle age (>=45 & <65)	224 (20.16)	79 (35.27)	145 (64.73)	
Older (>=65)	267 (64.27)	114 (42.7)	153 (57.3)	
Horvath clock (years)	58.37 (45.79; 68.73)	62.32 (50.58; 72.74)	56.06 (43.28; 66.68)	0.000
Hannum clock (years)	48.62 (36.67; 59.82)	53.68 (40.01; 63.39)	46.43 (34.58; 57.48)	0.000
PhenoAge (years)	43.75 (30.31; 55.76)	48.24 (33.12; 60.19)	41.88 (27.42;53.37)	0.000
DNAmGrimAge (years)	59.22 (47.44; 69.52)	64.55 (50.50; 75.21)	56.18 (45.17; 66.55)	0.000
iAge (years)	55.96 (50.19; 63.53)	56.27 (50.49; 64.18)	55.70 (50.05; 63.31)	0.327
BMI (kg/m^2)	24.24 (21.79; 27.07)	25.51 (23.05; 28.07)	23.50 (21.23; 26.40)	0.000
Total body fat (%)	31.57 (25.31; 37.61)	26.58 (21.18; 31.52)	35.07 (29.07; 40.13)	0.000
Android fat (%)	4.50 (4.09; 4.89)	4.75 (4.47; 5.18)	4.28 (3.92; 4.70)	0.000
Android/Gynoid ratio	0.45 (0.32; 0.62)	0.66 (0.47; 0.82)	0.37 (0.27; 0.47)	0.000
Physical activity (MET-min/week)*	1089 (453; 2184)	1215 (495; 2436)	993 (396; 1923)	0.020
Income (n=657)				0.001
<1500€	63 (9.59)	21 (33.33)	42 (66.67)	
1500€ à 2800€	174 (26.48)	46 (26.44)	128 (73.56)	
2800 à 4200€	201 (30.59)	74 (36.82)	127 (63.18)	
≥4200€	190(28.92)	90 (47.37)	100 (52.63)	
Don’t know/respond	29 (4.41)	12 (41.38)	17 (58.62)	
Marital status (n=661)				0.000
Maried/couple	442 (66.87)	192 (43.44)	250 (56.56)	
Single/divorced/widower	219 (33.13)	53 (24.2)	166 (75.8)	
Education level (n=662)				0.504
No schooling/primary	18 (2.72)	9 (50.00)	9 (50.00)	
Middle/high school	134 (20.24)	50 (37.31)	84 (62.69)	
College or higher	510 (77.04)	186 (36.47)	324 (63.53)	
Medications taken				0.744
0	247 (37.20)	98 (39.68)	149 (60.32)	
1 to 2	227 (34.19)	81 (35.68)	146 (64.32)	
3 to 4	94 (14.16)	34 (36.17)	60 (63.83)	
5 or more	96 (14.46)	33 (34.38)	63 (65.62)	
Current diseases (n=657)				0.050
0	322 (49.01)	106 (32.92)	216 (67.08)	
1 to 2	230 (35.01)	89 (38.70)	141 (61.3)	
3 or more	105 (15.98)	48 (45.71)	57 (54.29)	

*Physical activity in leisure time and transport. Mann-Whitney and chi-squared (two-sided p-values)

Supplementary table 5 Results after inclusion of the interaction term "DP-sex-age category" in the full adjusted linear regression model

Age group	Sex						
	Male			Female			
	β	SE	p	β	SE	p	Interaction (p)
Biological age acceleration by Horvath's clock							
	Plant-based DP*						
Younger	-1.39	1.73	0.423	0.16	1.17	0.890	0.646
Middle aged	0.62	1.44	0.666	0.50	1.03	0.631	
Older	-0.61	1.22	0.617	1.44	1.06	0.177	
	Sugar and Fast food DP						
Younger	1.65	2.08	0.427	0.18	1.47	0.905	0.948
Middle aged	-0.32	1.55	0.835	-0.72	1.39	0.603	
Older	1.56	1.38	0.260	0.78	1.58	0.624	
	Sandwiches DP						
Younger	-1.88	2.238	0.399	-1.197	1.765	0.498	0.630
Middle aged	3.75	1.93	0.052	0.94	1.39	0.498	
Older	-0.42	1.35	0.755	-1.24	1.75	0.476	
	aMDS*						
Younger	-0.34	0.41	0.407	-0.11	0.26	0.677	0.698
Middle aged	0.07	0.46	0.884	0.03	0.22	0.891	
Older	0.30	0.32	0.344	0.01	0.24	0.982	
	aDASH						
Younger	-0.09	0.25	0.721	-0.09	0.14	0.541	0.749
Middle aged	-0.08	0.20	0.692	-0.02	0.13	0.886	
Older	-0.08	0.16	0.607	0.17	0.15	0.274	
Biological age acceleration by Hannum's clock							
	Plant-based DP						
Younger	-0.31	1.39	0.823	-0.02	0.93	0.982	0.165
Middle aged	2.13	1.15	0.065	-0.10	0.82	0.902	
Older	-1.30	0.98	0.185	0.06	0.85	0.947	
	Sugar and Fast food DP*						
Younger	0.70	1.64	0.671	-0.82	1.16	0.481	0.537
Middle aged	-0.53	1.46	0.718	-0.66	1.10	0.549	
Older	0.34	1.09	0.757	1.69	1.25	0.176	
	Sandwiches DP*						
Younger	-1.93	1.78	0.278	-0.95	1.40	0.499	0.952
Middle aged	-0.85	1.72	0.622	-0.74	1.11	0.504	
Older	-0.11	1.07	0.921	0.10	1.39	0.941	
	aMDS						
Younger	0.21	0.33	0.518	0.03	0.21	0.870	0.994
Middle aged	-0.03	0.36	0.930	-0.24	0.18	0.176	
Older	-0.04	0.25	0.872	-0.19	0.19	0.313	
	aDASH						
Younger	0.14	0.19	0.478	-0.04	0.11	0.731	0.603
Middle aged	0.05	0.15	0.725	-0.02	0.10	0.846	

Older	-0.16	0.13	0.196	-0.07	0.12	0.560	
Biological age acceleration by PhenoAge							
	Plant-based DP						
Younger	-1.96	2.00	0.325	-0.67	1.34	0.620	0.038
Middle aged	2.30	1.66	0.167	-1.48	1.19	0.214	
Older	-3.26	1.41	0.021[a]	-0.11	1.22	0.927	
	Sugar and Fast food DP						
Younger	0.35	2.40	0.885	1.46	1.70	0.390	0.627
Middle aged	1.11	1.79	0.534	0.51	1.61	0.754	
Older	-0.24	1.60	0.883	2.44	1.82	0.184	
	Sandwiches DP						
Younger	1.77	2.59	0.494	-0.90	2.04	0.661	0.330
Middle aged	2.06	2.23	0.358	-1.05	1.61	0.517	
Older	-3.33	1.56	0.033[b]	-1.36	2.03	0.504	
	aMDS*						
Younger	0.06	0.50	0.899	0.21	0.30	0.471	0.870
Middle aged	-0.42	0.53	0.429	-0.46	0.26	0.072	
Older	-0.46	0.37	0.210	-0.12	0.28	0.664	
	aDASH						
Younger	-0.15	0.29	0.606	-0.07	0.17	0.674	0.681
Middle aged	-0.09	0.23	0.712	-0.14	0.15	0.347	
Older	-0.36	0.19	0.059	-0.09	0.18	0.620	
Biological age acceleration by GrimAge							
	Plant-based DP						
Younger	-1.57	1.24	0.208	0.90	0.84	0.282	0.389
Middle aged	0.67	1.03	0.515	0.46	0.74	0.537	
Older	-0.82	0.88	0.353	0.24	0.76	0.751	
	Sugar and Fast food DP						
Younger	2.30	1.49	0.123	0.09	1.05	0.931	0.441
Middle aged	-0.73	1.11	0.513	-0.76	1.00	0.448	
Older	-1.35	0.99	0.175	-0.57	1.14	0.615	
	Sandwiches DP						
Younger	-0.65	1.60	0.686	1.85	1.27	0.146	0.010
Middle aged	1.24	1.38	0.367	-1.58	1.00	0.114	
Older	-2.16	0.96	0.026[c,d,e]	1.86	1.25	0.137	
	aMDS						
Younger	0.02	0.29	0.958	0.24	0.18	0.185	0.242
Middle aged	-0.41	0.32	0.203	0.04	0.16	0.795	
Older	0.32	0.23	0.162	0.04	0.17	0.812	
	aDASH						
Younger	-0.28	0.18	0.121	-0.01	0.10	0.939	0.743
Middle aged	-0.11	0.14	0.416	-0.05	0.09	0.609	
Older	-0.09	0.12	0.434	0.08	0.11	0.438	
Biological age acceleration by iAge							
	Plant-based DP*						
Younger	8.15	3.79	0.032[f]	1.83	2.24	0.416	0.225
Middle aged	-0.75	2.81	0.789	0.03	1.99	0.986	

Older	-2.99	2.37	0.209	-0.08	2.07	0.969	
	Sugar and Fast food DP						
Younger	2.69	4.00	0.501	2.28	2.83	0.421	
Middle aged	0.43	3.08	0.890	-3.53	2.72	0.195	0.827
Older	1.77	2.67	0.506	-2.10	3.07	0.495	
	Sandwiches DP						
Younger	2.16	4.33	0.618	1.40	3.42	0.683	
Middle aged	0.47	3.74	0.901	-1.90	2.71	0.483	0.682
Older	-2.47	2.63	0.348	0.50	3.41	0.882	
	aMDS						
Younger	2.36	0.80	0.003[g,h,I,j]	0.25	0.50	0.610	
Middle aged	-0.36	0.91	0.690	-0.27	0.43	0.533	0.278
Older	0.85	0.64	0.184	-0.25	0.47	0.599	
	aDASH						
Younger	0.40	0.48	0.402	0.25	0.28	0.364	
Middle aged	0.09	0.40	0.824	-0.16	0.25	0.517	0.983
Older	-0.14	0.32	0.669	-0.23	0.30	0.433	

The linear regression models were adjusted for total medications, current diseases, educational level, income range, physical activity in leisure time and transport, and BMI

*After removing one case that was influencing the model (inspection of Leverage vs. Cook's distance)

[a] Significantly different from the middle-aged group of men (p=0.011)

[b] Significantly different from the middle-aged group of men (p=0.049)

[c,d,e] Significantly different from the middle-aged group of men (p=0.044), and from the younger (p=0.012) and the older (p=0.010) group of women.

[f] Significantly different from the older group of men (p=0.013)

[g,h,I,j] Significantly different from the young group of females (p=0.025); significantly different from the middle-aged group of males (p=0.024); significantly different from the middle-aged group of females (p=0.004); significantly different from the group of older females (p=0.005)

Supplementary table 6 Results after inclusion of the interaction terms “DP-sex” and “DP-age categories” in the full adjusted linear regression model

Dietary pattern	Horvath			Hannum			PhenoAge			GrimAge			iAge		
Plant-based	β	SE	p	β	SE	p	β	SE	p	β	SE	p	β	SE	p
Younger	-0.05	0.98	0.962	0.16	0.78	0.840	-0.81	1.12	0.472	0.45	0.71	0.529	1.92	1.89	0.310
Middle aged	0.69	0.83	0.412	0.61	0.67	0.360	-0.27	0.96	0.775	0.65	0.61	0.282	0.02	1.63	0.991
Older	0.59	0.81	0.465	-0.54	0.65	0.403	-1.45	0.93	0.121	-0.18	0.59	0.755	-1.27	1.58	0.422
DP x age (p interaction)	0.828*			0.658			0.678			0.592			0.434		
Female sex	0.73	0.63	0.249	-0.09	0.51	0.856	-0.85	0.73	0.245	0.48	0.46	0.297	0.52	1.23	0.670
Male sex	-0.03	0.82	0.975	0.27	0.66	0.678	-0.91	0.95	0.334	-0.07	0.60	0.900	-0.77	1.60	0.631
DP x sex (p interaction)	0.464*			0.457			0.955			0.459			0.520		
Sugar and fast-food	β	SE	p	β	SE	p	β	SE	p	β	SE	p	β	SE	p
Younger	0.03	1.18	0.979	-0.70	0.93	0.457	0.65	1.37	0.634	0.21	0.86	0.808	3.86	2.28	0.091
Middle aged	-0.62	1.03	0.549	-0.60	0.86	0.484	0.96	1.19	0.421	-0.82	0.75	0.273	-2.37	2.04	0.245
Older	1.65	1.05	0.117	1.26	0.83	0.132	1.41	1.22	0.246	-0.34	0.77	0.663	-1.35	2.05	0.511
DP x age (p interaction)	0.274*			0.182*			0.911			0.658			0.097		
Sex female	0.50	0.85	0.560	-0.36	0.76	0.638	0.03	1.05	0.980	-0.93	0.66	0.162	2.80	1.79	0.118
Sex male	0.33	0.91	0.716	0.35	0.67	0.602	1.65	0.99	0.095	-0.02	0.62	0.980	-2.05	1.66	0.218
DP x sex (p interaction)	0.892*			0.478*			0.252			0.306			0.044		
Sandwiches	β	SE	p	β	SE	p	β	SE	p	β	SE	p	β	SE	p
Younger	-1.44	1.39	0.301	-1.29	1.11	0.245	0.16	1.60	0.920	0.94	1.01	0.352	1.50	2.70	0.577
Middle aged	1.85	1.14	0.104	0.18	0.91	0.840	0.03	1.31	0.981	-0.72	0.83	0.385	-1.20	2.21	0.589
Older	-0.84	1.15	0.465	-0.11	0.92	0.904	-2.75	1.32	0.038	0.20	0.89	0.825	-1.47	2.24	0.511
DP x age (p interaction)	0.120			0.568			0.233			0.433*			0.656		
Female sex	-0.66	0.96	0.493	-0.64	0.77	0.403	-0.27	1.16	0.814	-0.27	0.78	0.730	-1.15	1.97	0.559
Male sex	0.91	1.01	0.367	0.20	0.81	0.803	-1.43	1.11	0.196	0.29	0.70	0.678	-0.21	1.87	0.909
DP x sex (p interaction)	0.265			0.453			0.475			0.594*			0.732		
aMDS	β	SE	p	β	SE	p	β	SE	p	β	SE	p	β	SE	p
Younger	-0.14	0.22	0.534	0.12	0.18	0.506	0.28	0.26	0.270	0.21	0.16	0.189	0.87	0.43	0.044
Middle aged	0.08	0.21	0.717	-0.16	0.16	0.325	-0.46	0.24	0.055	-0.03	0.15	0.829	-0.12	0.41	0.768
Older	0.16	0.19	0.407	-0.12	0.15	0.448	-0.22	0.22	0.327	0.19	0.14	0.167	0.07	0.38	0.858
DP x age (p interaction)	0.587			0.455			0.092			0.436			0.199		
Female sex	0.18	0.22	0.426	-0.16	0.11	0.153	-0.17	0.16	0.284	0.09	0.10	0.347	-0.11	0.27	0.691
Male sex	-0.01	0.14	0.915	0.07	0.18	0.692	-0.19	0.25	0.464	0.15	0.16	0.329	0.74	0.44	0.092
DP x sex (p interaction)	0.465			0.269			0.965			0.749			0.100		
aDASH	β	SE	p	β	SE	p	β	SE	p	β	SE	p	β	SE	p
Younger	-0.03	0.12	0.827	0.05	0.10	0.594	-0.04	0.15	0.761	-0.02	0.09	0.799	0.11	0.24	0.654
Middle aged	-0.01	0.11	0.958	0.00	0.08	0.978	-0.15	0.13	0.242	-0.06	0.08	0.440	-0.03	0.21	0.873
Older	0.02	0.11	0.874	-0.13	0.09	0.120	-0.23	0.13	0.076	-0.04	0.08	0.626	-0.12	0.22	0.588
DP x age (p interaction)	0.964*			0.316			0.627			0.948			0.779		

Female sex	0.02	0.08	0.822	-0.06	0.06	0.326	-0.13	0.10	0.192	-0.01	0.06	0.903	-0.05	0.16	0.756
Male sex	-0.04	0.11	0.755	0.00	0.09	0.963	-0.20	0.13	0.136	-0.10	0.08	0.227	0.00	0.23	0.996
DP x sex (p interaction)	0.699*			0.583			0.671			0.366			0.852		

The linear regression models were adjusted for total medications, current diseases, educational level, income range, physical activity in leisure time and transport, and BMI
*After removing one case that was influencing the model (inspection of Leverage vs. Cook's distance)

Supplementary table 7 Results for the mediation analysis of the relationship between DP and biological age acceleration, with total body fat (%) as mediator

	Horvath β (95%CI)	p	Hannum β (95%CI)	p	PhenoAge β (95%CI)	p	GrimAge β (95%CI)	p	iAge β (95%CI)	p
Plant-based DP										
Path a	-2.37(-3.97; -0.77)	0.004	-2.36 (-3.97; -0.77)	0.004	-2.37 (-3.97; -0.77)	0.004	-2.39 (-3.99; -0.79)	0.003	-2.40 (-4.01; -0.80)	0.003
Path b	0.05 (-0.00; 0.10)	0.066	0.01 (-0.03; 0.05)	0.467	0.09 (0.03; 0.15)	0.003	0.03 (-0.00; 0.07)	0.066	-0.12 (-0.21; -0.02)	0.019
Direct effect	0.50 (-0.47;1.47)	0.314	0.07 (-0.71; 0.84)	0.866	-0.80 (-1.92; 0.32	0.160	0.22 (-0.50; 0.93)	0.551	-0.02 (-1.92; 1.88)	0.985
Indirect effect	-0.11 (-.27; 0.05)	0.169	-0.04 (-0.14; 0.07)	0.527	-0.21 (-0.41; -0.01)	0.044	-0.08 (-0.18; 0.02)	0.128	0.28 (-0.03; 0.59)	0.075
Total effect	0.39 (-0.58; 1.36)	0.431	0.03 (-0.74; 0.80)	0.935	-1.01 (-2.12; 0.11)	0.077	0.13 (-0.58; 0.84)	0.710	0.26 (-1.63; 2.15)	0.786
Sugar and Fast-food DP										
Path a	-0.25 (-2.29; 1.80)	0.811	-0.25 (-2.30; 1.80)	0.811	-0.25 (-2.30; 1.80)	0.811	-0.28 (-2.33; 1.76)	0.785	-0.19 (-2.25; 1.87)	0.858
Path b	0.04 (-0.01; 0.09)	0.083	0.01 (-0.03; 0.05)	0.481	0.09 (0.04; 0.15)	0.001	0.03 (-0.00; 0.07)	0.077	-0.12 (-0.21; -0.02)	0.018
Direct effect	0.41 (-0.82; 1.63)	0.516	-0.41 (-1.38; 0.57)	0.417	0.93 (-0.48; 2.34)	0.196	-0.43 (-1.33; 0.47)	0.350	0.12 (-2.28; 2.52)	0.923
Indirect effect	-0.01 (-0.12; 0.10)	0.845	-0.00 (-0.06; 0.05)	0.898	-0.02 (-0.23; 0.19)	0.827	-0.01 (-0.09; 0.07)	0.826	0.02 (-0.24; 0.29)	0.871
Total effect	0.39 (-0.83; 1.63)	0.529	-0.41 (-1.39; .57)	0.413	0.90 (-0.52; 2.33)	0.212	-0.44 (-1.34; 0.46)	0.341	0.14 (-2.27; 2.55)	0.909
Sandwiches DP										
Path a	0.09 (-2.13; 2.31)	0.938	0.09 (-2.13; 2.31)	0.938	0.09 (-2.13; 2.31)	0.938	0.15 (-2.06; 2.37)	0.890	0.04 (-2.18; 2.26)	0.973
Path b	0.04 (-0.01; 0.09)	0.084	0.01 (-0.03; 0.05)	0.476	0.09 (0.04; 0.15)	0.001	0.03 (-0.00; 0.06)	0.076	-0.12 (-0.21; -0.02)	0.018
Direct effect	0.20 (-1.13; 1.53)	0.765	-0.19 (-1.25; 0.88)	0.731	-0.80 (-2.32; 0.73)	0.309	-0.07 (-1.04; 0.91)	0.895	-0.95 (-3.53; 1.63)	0.470
Indirect effect	0.00 (-0.11; 0.11)	0.945	0.00 (-0.05; 0.05)	0.963	0.01 (-0.19; 0.21)	0.936	0.01 (-0.08; 0.09)	0.902	-0.00 (-0.28; 0.27)	0.975
Total effect	0.21 (-1.13; 1.54)	0.762	-0.19 (-1.25; 0.88)	0.733	-0.78 (-2.32; 0.76)	0.319	-0.06 (-1.04; 0.92)	0.903	-0.96 (-3.55; 1.64)	0.470
aMDS										
Path a	-0.50 (-0.88; -0.12)	0.009	-0.50 (-0.87; -0.12)	0.009	-0.50 (-0.88; -0.12)	0.009	-0.51 (-0.88; -0.13)	0.008	-0.49 (-0.87; -0.11)	0.011
Path b	0.04 (-0.01; 0.09	0.090	0.02 (0.02; 0.05)	0.422	0.08 (0.03; 0.14)	0.003	0.04 (0.00; 0.07)	0.026	-0.10 (-0.20; -0.01)	0.029

Direct effect	0.04 (-0.19; 0.27)	0.744	-0.08 (-0.26; 0.10)	0.378	-0.14 (-0.41; 0.12)	0.284	0.11 (-0.05; 0.27)	0.202	0.15 (-0.31; 0.60)	0.527
Indirect effect	-0.02 (-0.04; 0.01)	0.146	-0.01 (-0.03; 0.01)	0.487	-0.04 (-0.08; -0.00)	0.043	-0.02 (-0.04; 0.00)	0.099	0.05 (-0.01; 0.11)	0.103
Total effect	0.02 (-0.21; 0.25)	0.881	-0.09 (-0.27; 0.09)	0.332	-0.19 (-0.45; 0.08)	0.168	0.09 (-0.08; 0.25)	0.297	0.20 (-0.25; 0.65)	0.392
aDASH										
Path a	-0.25 (-0.47; -0.04)	0.019	-0.25 (-0.47; -0.04)	0.019	-0.25 (-0.47; -0.04)	0.019	-0.25 (-0.46; -0.04)	0.021	-0.28 (-0.50; -0.07)	0.009
Path b	0.04 (-0.01; 0.09)	0.122	0.02 (-0.01; 0.06)	0.230	0.07 (0.02; 0.13)	0.010	0.03 (-0.00; 0.06)	0.075	-0.11 (-0.21; -0.02)	0.012
Direct effect	-0.01 (-0.14; 0.12)	0.876	-0.03 (-0.13; 0.07)	0.532	-0.14 (-0.29; 0.01)	0.071	-0.04 (-0.14; 0.05)	0.367	-0.04 (-0.29; 0.22)	0.767
Indirect effect	-0.01 (-0.02; 0.01)	0.214	-0.01 (-0.02; 0.01)	0.343	0.02 (-0.04; 0.00)	0.090	-0.01 (-0.02; 0.00)	0.202	0.03 (-0.00; 0.07)	0.070
Total effect	-0.02 (-0.15; 0.11)	0.764	-0.03 (-0.13; 0.06)	0.460	-0.16 (-0.31; -0.01)	0.041	-0.05 (-0.15; 0.04)	0.286	-0.00 (-0.26; 0.25)	0.972

Path a: dietary pattern → %body fat
Path b: %body fat → biological age acceleration
Direct effect (c'): dietary pattern → biological age acceleration
Indirect effect (bootstrapped) (a x b): dietary pattern →%body fat → biological age acceleration
Total effect: indirect + direct effect (c' + ab)
Two-sided z-test
No adjustments were made for multiple comparisons

Supplementary table 8 Results for the mediation analysis of the relationship between DP and biological age acceleration, with android fat (%) as mediator

	Horvath β (95%CI)	p	Hannum β (95%CI)	p	PhenoAge β (95%CI)	p	GrimAge β (95%CI)	p	iAge β (95%CI)	**p**
Plant-based DP										
Path a	0.26 (0.14; 0.39)	0.000	0.26 (0.14; 0.39)	0.000	0.26 (0.14; 0.39)	0.000	0.26 (0.14; 0.39)	0.000	0.26 (0.14; 0.38)	0.000
Path b	-0.44 (-1.09; 0.20)	0.178	-0.03 (-0.54; 0.48)	0.896	-0.58 (-1.32; 0.16)	0.125	-0.10 (-0.57; 0.37)	0.681	1.17 (-0.08; 2.43)	0.068
Direct effect	0.51 (-0.48; 1.49)	0.313	0.04 (-0.74; 0.83)	0.918	-0.86 (-1.99; 0.28)	0.138	0.16 (-0.56; 0.88)	0.662	-0.04 (-1.96; 1.87)	0.965
Indirect	-0.12 (-0.31; 0.08)	0.242	-0.01 (-0.14; 0.13)	0.900	-0.15 (-0.38; 0.07)	0.181	-0.02 (-0.16; 0.11)	0.715	0.31 (-0.06; 0.68)	0.105
Total effect	0.39 (-0.58; 1.36)	0.431	0.03 (-0.74; 0.80)	0.935	-1.01 (-2.12; 0.11)	0.077	0.13 (-0.57; 0.84)	0.710	0.26 (-1.63; 2.15)	0.786
Sugar and Fast-food DP										
Path a	-0.02 (-0.18; 0.14)	0.810	-0.02 (-0.18; 0.14)	0.810	-0.02 (-0.18; 0.14)	0.810	-0.02 (-0.17; 0.14)	0.809	-0.02 (-0.18; 0.14)	0.835
Path b	-0.38 (-1.02; 0.25)	0.236	-0.03 (-0.54; 0.47)	0.902	-0.68 (-1.41; 0.06)	0.071	-0.08 (-0.55; 0.38)	0.726	1.17 (-0.07; 2.41)	0.065
Direct effect	0.39 (-0.84; 1.62)	0.536	-0.41 (-1.39; 0.57)	0.412	0.89 (-0.52; 2.31)	0.217	-0.44 (-1.34; 0.46)	0.339	0.16 (-2.25; 2.57)	0.896
Indirect effect	0.01 (-0.06; 0.08)	0.833	0.00 (-0.03; 0.04)	0.974	0.01 (-0.11; 0.13)	0.832	0.00 (-0.04; 0.04)	0.935	-0.02 (-0.23; 0.19)	0.851
Total effect	0.40 (-0.83; 1.63)	0.529	-0.41 (-1.39; 0.57)	0.413	0.90 (-0.51; 2.33)	0.212	-0.44 (-1.33; 0.46)	0.341	0.14 (-2.27; 2.55)	0.909
Sandwiches DP										
Path a	0.00 (-0.16; 0.18)	0.960	0.00 (-0.16; 0.17)	0.960	0.00 (-0.17; 0.18)	0.960	-0.00 (-0.17; 0.17)	0.997	0.01 (-0.16; 0.18)	0.906
Path b	-0.39 (-1.02; 0.25)	0.234	-0.03 (-0.53; 0.48	0.909	-0.67 (-1.41; 0.05)	0.070	-0.08 (-0.55; 0.38)	0.733	1.17 (-0.07; 2.41)	0.065
Direct effect	0.21 (-1.12; 1.54)	0.759	-0.18 (-1.25; 0.88)	0.733	-0.78 (-2.32; 0.75)	0.319	-0.06 (-1.04; 0.92)	0.903	-0.97 (-3.55; 1.62)	0.463
Indirect effect	-0.00 (-0.09; 0.09)	0.971	-0.00 (-0.05; 0.04)	0.996	-0.00 (-0.14; 0.13)	0.965	0.00 (-0.04; 0.04)	0.999	0.01 (-0.22; 0.24)	0.917
Total effect	0.21 (-1.13; 1.54)	0.762	-0.19 (-1.24; 0.88)	0.733	-0.78 (-2.32; 0.76)	0.319	-0.06 (-1.04; 0.92)	0.903	-0.95 (-3.55; 1.64)	0.470
aMDS										
Path a	-0.06 (-0.12; -0.01)	0.026	-0.06 (-0.12; -0.01)	0.026	-0.06 (-0.12; -0.01)	0.026	-0.06 (-0.11; -0.01)	0.024	-0.06 (-0.12; -0.01)	0.029

Path b	0.29 (-0.03; 0.62)	0.078	0.06 (-0.19; 0.32)	0.634	0.66 (0.29; 1.03)	0.000	0.31 (0.08; 0.54)	0.009	-0.77 (-1.40; -0.13)	0.018
Direct effect	0.04 (-0.19; 0.27)	0.759	-0.09 (-0.26; 0.10)	0.355	-0.14 (-0.40; 0.12)	0.282	0.11 (-0.06; 0.27)	0.201	0.15 (-0.30; 0.60)	0.517
Indirect effect	-0.02 (-0.05; 0.01)	0.186	-0.00 (-0.02; 0.01)	0.672	-0.04 (-0.09; 0.00)	0.062	-0.02 (-0.04; 0.00)	0.095	0.05 (-0.01; 0.11)	0.129
Total effect	0.02 (-0.21; 0.25)	0.881	-0.09 (-0.27; 0.09)	0.332	-0.19 (-0.45; 0.08)	0.168	0.09 (-0.07; 0.25)	0.297	0.20 (-0.25; 0.65)	0.392
aDASH										
Path a	-0.04 (-0.07; -0.01)	0.008	-0.04 (-0.07; -0.1)	0.008	-0.04 (-0.07; -0.01)	0.008	-0.04 (-0.07; -0.01)	0.009	-0.05 (-0.08; -0.02)	0.004
Path b	0.29 (-0.03; 0.61)	0.077	0.10 (-0.15; 0.34)	0.449	0.59 (0.21; 0.96)	0.002	0.25 (0.02; 0.48)	0.036	-0.87 (-1.50; -0.25)	0.006
Direct effect	-0.01 (-0.14; 0.12)	0.909	-0.03 (-0.13; 0.07)	0.512	-0.13 (-0.28; 0.02)	0.085	-0.04 (-0.13; 0.05)	0.397	-0.05 (-0.30; 0.21)	0.726
Indirect effect	-0.01 (-0.03; 0.01)	0.169	-0.00 (-0.01; 0.01)	0.489	-0.03 (-0.05; -0.00)	0.040	-0.01 (-0.02; 0.00)	0.119	0.04 (0.00; 0.08)	0.048
Total effect	-0.02 (-0.15; 0.11)	0.764	-0.04 (-0.14; 0.06)	0.460	-0.16 (-0.31; -0.01)	0.041	-0.05 (-0.15; 0.04)	0.286	-0.00 (-0.26; 0.25)	0.972

Path a: dietary pattern → %android fat
Path b: %android fat → biological age acceleration
Direct effect (c’): dietary pattern → biological age acceleration
Indirect effect (bootstrapped) (a x b): dietary pattern →%android fat → biological age acceleration
Total effect: indirect + direct effect (c’ + ab)
Two-sided z-test
No adjustments were made for multiple comparisons

Supplementary table 9 Results for the mediation analysis of the relationship between DP and biological age acceleration, with the android-to-gynoid fat ratio as mediator

	Horvath β (95%CI)	p	Hannum β (95%CI)	p	Levine β (95%CI)	p	GrimAge β (95%CI)	p	iAge β (95%CI)	p
Plant-based DP										
Path a	-0.04 (-0.08; -0.00)	0.029	-0.04 (-0.08; -0.00)	0.029	-0.04 (-0.08; -0.00)	0.029	-0.04 (-0.07; -0.00)	0.029	-0.04 (-.08; -0.00)	0.030
Path b	1.57 (-0.64; 3.78)	0.163	-0.11 (-1.86; 1.65)	0.906	3.06 (0.53; 5.59)	0.018	2.19 (0.58; 3.80)	0.008	-4.97 (-9.27; -0.67)	0.023
Direct effect (c’)	0.45 (-0.52; 1.42)	0.361	0.03 (-0.74; 0.80)	0.944	-0.88 (-2.00; 0.23)	0.121	0.22 (-0.48; 0.93)	0.536	0.06 (-1.83; 1.95)	0.949
Indirect effect (a x b)	-0.06 (-0.19; 0.06)	0.331	0.00 (-0.07; 0.08)	0.912	-0.12 (-0.30; 0.05)	0.162	-0.09 (-0.20; 0.02)	0.0126	0.20 (-0.08; 0.48)	0.159
Total effect (c’ + ab)	0.39 (-0.58; 1.36)	0.431	0.03 (-0.74; 0.80)	0.935	-1.01 (-2.13; 0.11)	0.077	0.13 (-0.57; 0.84)	0.710	0.26 (-1.63; 2.15)	0.786
Sugar and Fast-food DP										
Path a	-0.01 (-.06; 0.03)	0.604	-0.01 (-0.06; 0.03)	0.604	-0.01 (-0.06; 0.03)	0.604	-0.01 (-0.06; 0.03)	0.592	-0.01 (-0.06; 0.03)	0.608
Path b	1.49 (-0.77; 3.68)	0.184	-0.13 (-1.88; 1.62)	0.886	3.28 (0.76; 5.80)	0.011	2.13 (0.53; 3.73)	0.009	-4.98 (-9.26; -0.69)	0.023
Direct effect	0.41 (-0.81; 1.64)	0.509	-0.41 (-1.39; 0.57)	0.411	0.94 (-0.47; 2.36)	0.190	-0.41 (-1.31; 0.49)	0.369	0.08 (-2.32; 2.48)	0.948
Indirect effect	-0.02 (-0.11; 0.07)	0.696	0.00 (-0.04; 0.04)	0.941	-0.04 (-0.22; 0.14)	0.659	-0.03 (-0.14; 0.08)	0.638	0.06 (-0.22; 0.35)	0.677
Total effect	0.40 (-0.83; 1.63)	0.529	-0.41 (-1.39; 0.57)	0.413	0.90 (-0.52; 2.33)	0.212	-0.44 (-1.34; 0.46)	0.341	0.14 (-2.27; 2.55)	0.909
Sandwiches DP										
Path a	.03 (-0.02; 0.08)	0.297	0.03 (-0.02; 0.08)	0.297	0.03 (-0.02; 0.08)	0.297	0.03 (-0.02; 0.08)	0.307	0.03 (-0.02; 0.07)	0.320
Path b	1.46 (-0.74; 3.65)	0.193	-0.10 (-1.85; 1.65)	0.912	3.31 (0.78; 5.83)	0.010	2.15 (0.55; 3.75)	0.008	-4.93 (-9.21; -0.64)	0.024
Direct effect	0.17 (-1.16; 1.50)	0.805	-0.18 (-1.25; 0.88)	0.737	-0.87 (-2.40; 0.66)	0.265	-0.12 (-1.09; 0.86)	0.813	-0.83 (-3.42; 1.75)	0.528
Indirect effect	0.04 (-0.07; 0.15)	0.488	-0.00 (-0.07; 0.07)	0.940	0.09 (-0.11; 0.29)	0.383	0.06 (-0.08; 0.20)	0.433	-0.13 (-0.45; 0.20)	0.447
Total effect	0.21 (-1.13; 1.54)	0.762	-0.19 (-1.25; 0.88)	0.733	-0.78 (-2.32; 0.76)	0.319	-0.06 (-1.04; 0.92)	0.903	-0.96 (-3.55; 1.64)	0.470
aMDS										
Path a	-0.00 (-0.01; 0.00)	0.291	-0.00 (-0.01; 0.00)	0.291	-0.00 (-0.01; 0.00)	0.291	-0.00 (-0.01; 0.00)	0.293	-0.00 (-0.01; 0.00)	0.302

Path b	1.44 (-0.63; 3.51)	0.172	0.02 (-1.61; 1.66)	0.977	3.18 (0.81; 5.55)	0.008	2.16 (0.68; 3.63)	0.004	-4.91 (-8.95; -0.88)	0.017
Direct effect	0.02 (-0.21; 0.25)	0.835	-0.09 (-0.27; 0.09)	0.333	-0.17 (-0.43; 0.09)	0.203	0.09 (-0.07; 0.26)	0.242	0.17 (-0.27; 0.62)	0.448
Indirect effect	-0.01 (-0.03; 0.01)	0.482	-0.00 (-0.01; 0.01)	0.983	-0.01 (-0.05; 0.02)	0.381	-0.01 (-0.03; 0.01)	0.348	0.02 (-0.03; 0.08)	0.427
Total effect	0.02 (-0.21; 0.25)	0.881	-0.09 (-0.27; 0.09)	0.332	-0.19 (-0.45; 0.08)	0.168	0.09 (-0.08; 0.25)	0.297	0.20 (-0.25; 0.65)	0.392
aDASH										
Path a	-0.01 (-0.01; -0.00)	0.036	-0.01 (-0.01; -0.00)	0.036	-0.01 (-0.01; -0.00)	0.036	-0.01 (-0.01; -0.00)	0.037	-0.01 (-0.01; -0.00)	0.030
Path b	1.88 (-0.16; 3.92)	0.071	0.16 (-1.40; 1.72)	0.845	3.18 (0.80; 5.56)	0.009	1.69 (0.23; 3.16)	0.023	-5.37 (-9.33; -1.41)	0.008
Direct effect	-0.01 (-0.14; 0.12)	0.881	-0.04 (-0.13; 0.06)	0.472	-0.14 (-0.29; 0.01)	0.067	-.04 (-0.14; 0.05)	0.380	-0.03 (-0.29; 0.22)	0.791
Indirect effect	-0.01 (-0.03; 0.01)	0.200	-0.00 (-0.01; 0.01)	0.868	-0.01 (-0.04; 0.01)	0.140	-0.01 (-0.02; 0.00)	0.158	0.03 (-0.01; 0.07)	0.121
Total effect	-0.02 (-0.15; 0.11)	0.764	-0.04 (-0.14; 0.06)	0.460	-0.16 (-0.31; -0.01)	0.041	-0.05 (-0.14; 0.04)	0.286	-0.00 (-0.25; 0.25)	0.972

Path a: dietary pattern → android/gynoid fat
Path b: android/gynoid fat → biological age acceleration
Direct effect (c'): dietary pattern → biological age acceleration
Indirect effect (bootstrapped) (a x b): dietary pattern→ android/gynoid→ biological age acceleration
Total effect: indirect + direct effect (c' + ab)
Two-sided z-test
No adjustments were made for multiple comparisons

Supplementary table 10 Regression analysis for the association between the complete MDS (with alcohol, 0 to 9) and the biological age acceleration according to epigenetic and inflammation clocks

Biological clock	Model 1 β (95%CI) N=238	p	Model 2 β (95%CI) N=226	p	Model 3 β (95%CI) N=209	p
Horvath's clock	-0.22 (-0.58; 0.16)	0.255	-0.18 (-0.55; 0.18)	0.327	-0.11 (-0.51; 0.28)	0.567
Hannum's clock	-0.03 (-0.31; 0.26)	0.849	-0.01 (-0.30; 0.27)	0.921	-0.02 (-0.34; 0.28)	0.856
PhenoAge	-0.19 (-0.62; 0.24)	0.386	-0.17 (-0.62; 0.27)	0.438	-0.10 (-0.56; 0.36)	0.665
GrimAge	0.25 (0.00; 0.49)	0.047	0.24 (0.00; 0.48)	0.046	0.30 (0.05; 0.55)	0.019
iAge	-0.15 (-0.80; 0.51)	0.663	-0.17 (-0.88; 0.54)	0.631	-0.25 (-1.01; 0.50)	0.511

Model 1: unadjusted
Model 2: age categories, sex, total medications, current diseases, educational level, income range, and physical activity in leisure time and transport
Model 3: Model 2 + BMI

Supplementary table 11 Results after inclusion of the interaction term "DP-sex-age categories" in the linear regression for the complete aMDS (with alcohol, 0 to 9)

Age group	Male			Female		
	β	SE	p	β	SE	p
Horvath's clock						
Younger	-0.22	0.62	0.725	-0.24	0.63	0.699
Middle aged	0.18	0.88	0.833	-0.19	0.36	0.602
Older	0.08	0.46	0.865	-0.22	0.41	0.596
Hannum's clock						
Younger	0.13	0.48	0.795	0.14	0.49	0.769
Middle aged	-0.33	0.68	0.630	-0.24	0.28	0.406
Older	0.14	0.36	0.693	-0.10	0.32	0.765
PhenoAge						
Younger	-0.13	0.73	0.863	-0.01	0.74	0.991
Middle aged	-0.73	1.04	0.485	0.30	0.43	0.487
Older	-0.05	0.54	0.927	-0.66	0.49	0.181
GrimAge						
Younger	-0.13	0.38	0.741	0.84	0.47	0.076
Middle aged	-0.34	0.54	0.523	0.44	0.22	0.049
Older	0.47	0.28	0.094	0.21	0.25	0.412
iAge						
Younger	0.82	1.14	0.477	1.68	1.16	0.149
Middle aged	0.89	1.62	0.584	-1.45	0.67	0.032[a,b]
Older	1.72	0.87	0.048	-1.73	0.77	0.025[c,d]

*Exclusion of one participant after inspection of Leverage vs. Cook's distance
[a,b] Significantly different in relation to the group of young females (p=0.020); significantly different in relation to the older group of males (p=0.005)
[c,d] Significantly different in relation to the young group of females (p=0.015); significantly different in relation to the older group of males (p=0.003)
Two-sided t-test
No adjustments were made for multiple comparisons

Supplementary table 12 Mediation analysis for the complete aMDS (with alcohol, 0 to 9) and biological age acceleration

MDS	Horvath β (95%CI)	p	Hannum β (95%CI)	p	PhenoAge β (95%CI)	p	GrimAge β (95%CI)	p	iAge β(95%CI)	p
Total body fat (%)										
Path a	-0.41 (-0.98; 0.15)	0.152	-0.41 (-0.98; 0.15)	0.152	-0.41 (-0.98; 0.15)	0.152	-0.43 (-0.99; 0.13)	0.136	-0.41 (-0.98; 0.16)	0.157
Path b	0.00 (-0.08; 0.09)	0.915	0.05 (-0.02; 0.12)	0.129	0.04 (-0.06; 0.15)	0.454	0.06 (0.00; 0.12)	0.040	-0.11 (-0.29; 0.06)	0.194
Direct effect	-0.11 (-0.49; 0.26)	0.561	-0.02 (-0.31; 0.27)	0.888	-0.12 (-0.56; 0.33)	0.596	0.29 (0.04; 0.53)	0.021	-0.24 (-0.97; 0.48)	0.508
Indirect effect	-0.00 (-.005; 0.05)	0.934	-0.02 (-0.07; 0.03)	0.399	-0.02 (-.07; 0.04)	0.564	-0.03 (-0.07; 0.02)	0.285	0.05 (-0.07; 0.17)	0.450
Total effect	-0.11 (-0.48; 0.26)	0.552	-0.04 (-0.34; 0.25)	0.772	-0.14 (-0.58; 0.31)	0.544	0.26 (0.01; 0.50)	0.036	-0.20 (-0.92; 0.52)	0.592
Android fat (%)										
Path a	-0.04 (-0.12; 0.05)	0.368	-0.04 (-0.13; 0.05)	0.368	-0.04 (-0.12; 0.05)	0.368	-0.04 (-0.13; 0.05)	0.341	-0.04 (-0.13; 0.05)	0.369
Path b	0.07 (-0.50; 0.64)	0.801	0.29 (-0.16; 0.74)	0.209	0.48 (-0.20; 1.16)	0.164	0.35 (-0.02; 0.72)	0.065	-0.87 (-1.96; 0.23)	0.120
Direct effect	-0.11 (-0.48; 0.26)	0.563	-0.03 (-0.32; 0.26)	0.832	-0.12 (-0.56; 0.33)		0.27 (0.03; 0.52)	0.026	-0.23 (-.95; 0.49)	0.526
Indirect effect	-0.00 (-0.04; 0.03)	0.867	-0.01 (-0.05; 0.03)	0.574	-0.02 (-0.08; 0.04)	0.548	-0.01 (-0.05; 0.02)	0.455	0.04 (-0.09; 0.16)	0.571
Total effect	-0.00 (-0.03; 0.02)	0.808	-0.04 (-0.34; 0.25)	0.772	-0.14 (-0.58; 0.31)	0.544	0.26 (0.02; 0.50)	0.036	-0.20 (-0.92; 0.54)	0.592
Android/gynoid ratio										
Path a	-0.00 (-0.02; 0.01)	0.824	-0.00 (-0.02; 0.01)	0.824	-0.00 (-0.02; 0.01)	0.824	-0.00 (-0.02; 0.01)	0.817	-0.00 (-0.02; 0.01)	0.819
Path b	0.34 (-3.26; 3.94)	0.853	0.69 (-2.15; 3.53)	0.635	2.89 (-1.40; 7.18)	0.187	1.88 (-0.44; 4.21)	0.113	-6.44 (-13.3; 0.45)	0.067
Direct effect	-0.11 (-0.49; 0.26)	0.554	-0.04 (-0.34; 0.25)	0.777	-0.13 (-0.58; 0.31)	0.556	0.26 (0.02; 0.50)	0.033	-0.21 (-0.92; 0.51)	0.569
Indirect effect	-0.00 (-0.03; 0.03)	0.968	-0.00 (-0.03; 0.03)	0.938	-0.00 (-0.06; 0.06)	0.886	-0.00 (-0.04; 0.03)	0.860	0.01 (-0.13; 0.15)	0.880
Total effect	-0.11 (-0.48; 0.26)	0.552	-0.04 (-0.34; 0.25)	0.772	-0.14 (-0.58; 0.31)	0.544	0.26 (0.02; 0.50)	0.036	-0.20 (-0.92; 0.52)	0.592

Path a: dietary pattern → %total body fat or %android fat or android/gynoid. Path b: %total body fat or %android fat or android/gynoid → biological age acceleration. Direct effect (c'): dietary pattern → biological age acceleration. Indirect effect (bootstrapped) (a x b): dietary pattern → body fat → biological age acceleration. Total effect: indirect + direct effect (c' + ab)
Two-sided t-test
No adjustments were made for multiple comparisons

Supplementary figures: plots for visually inspecting linearity, normality, and homocedasticity for the associations between dietary patterns (DPs) and biological clocks

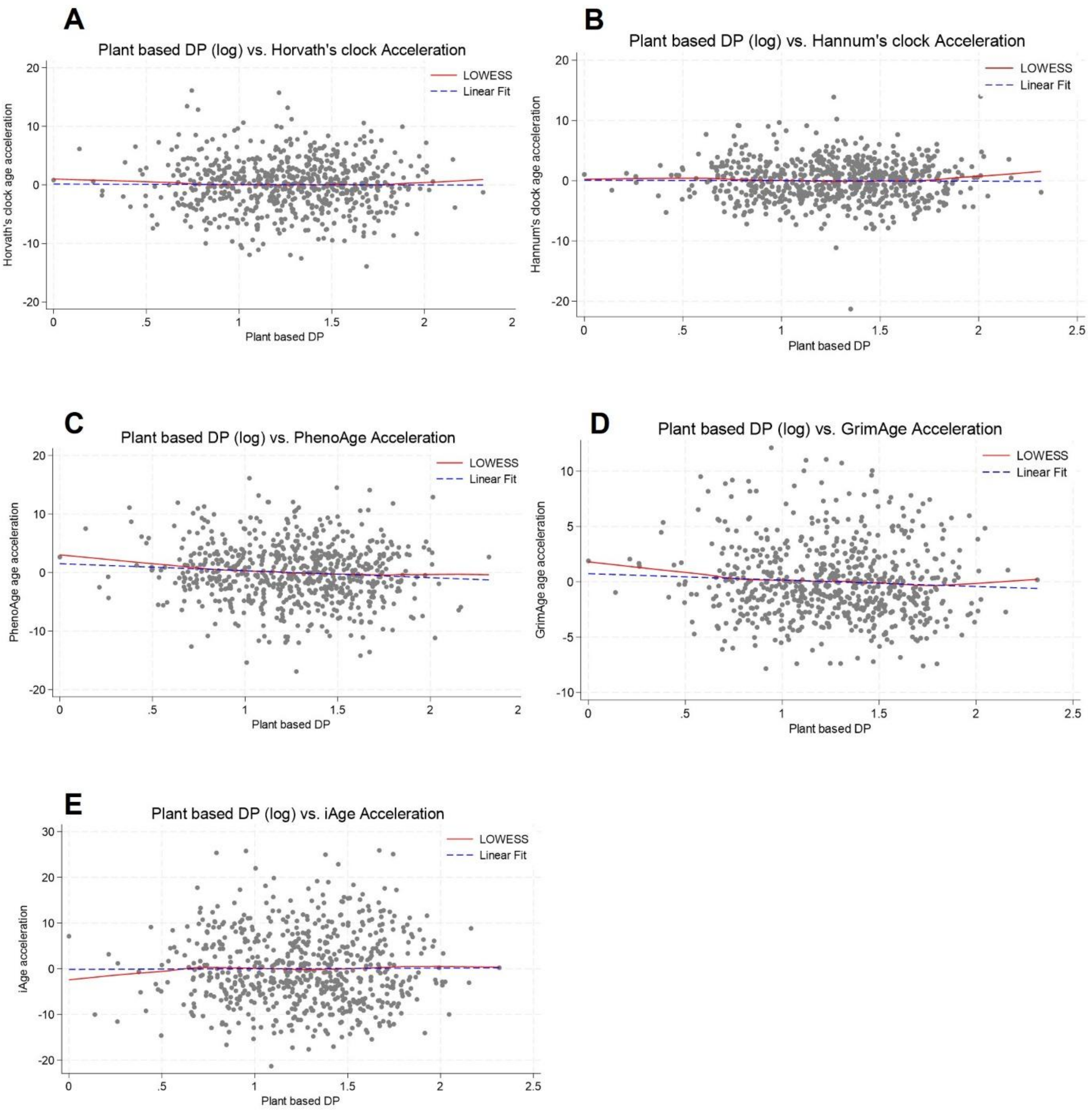

Supplementary figure 1 Scatterplots for visually inspecting linearity between the “Plant-based” dietary pattern (DP) and biological age acceleration according to A – Horvath’s clock, B – Hannum’s clock, C – PhenoAge, D – GrimAge, E – iAge

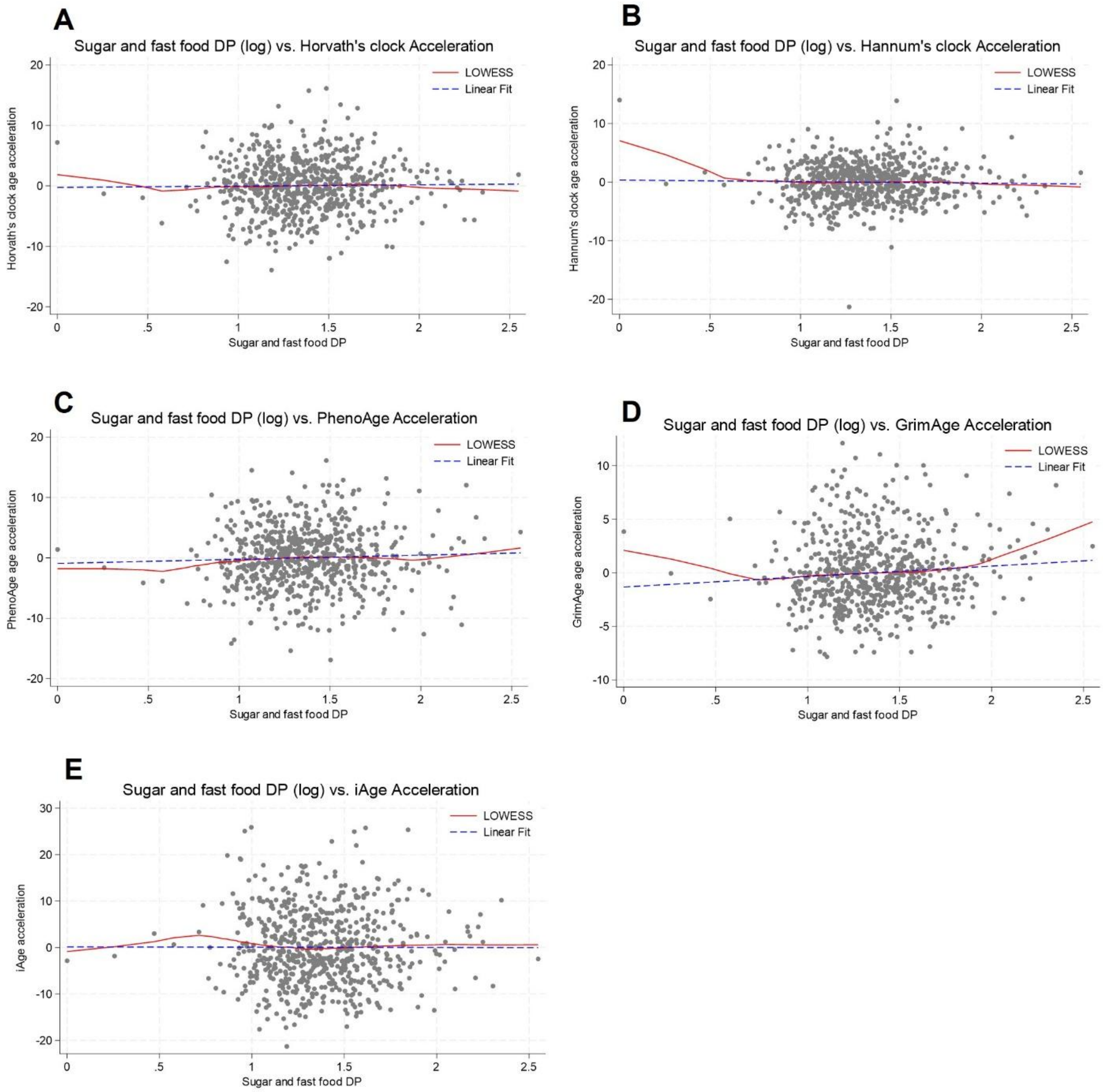

Supplementary figure 2 Scatterplots for visually inspecting linearity between the "Sugar and fast food" dietary pattern (DP) and biological age acceleration according to A – Horvath's clock, B – Hannum's clock, C – PhenoAge, D – GrimAge, E – iAge

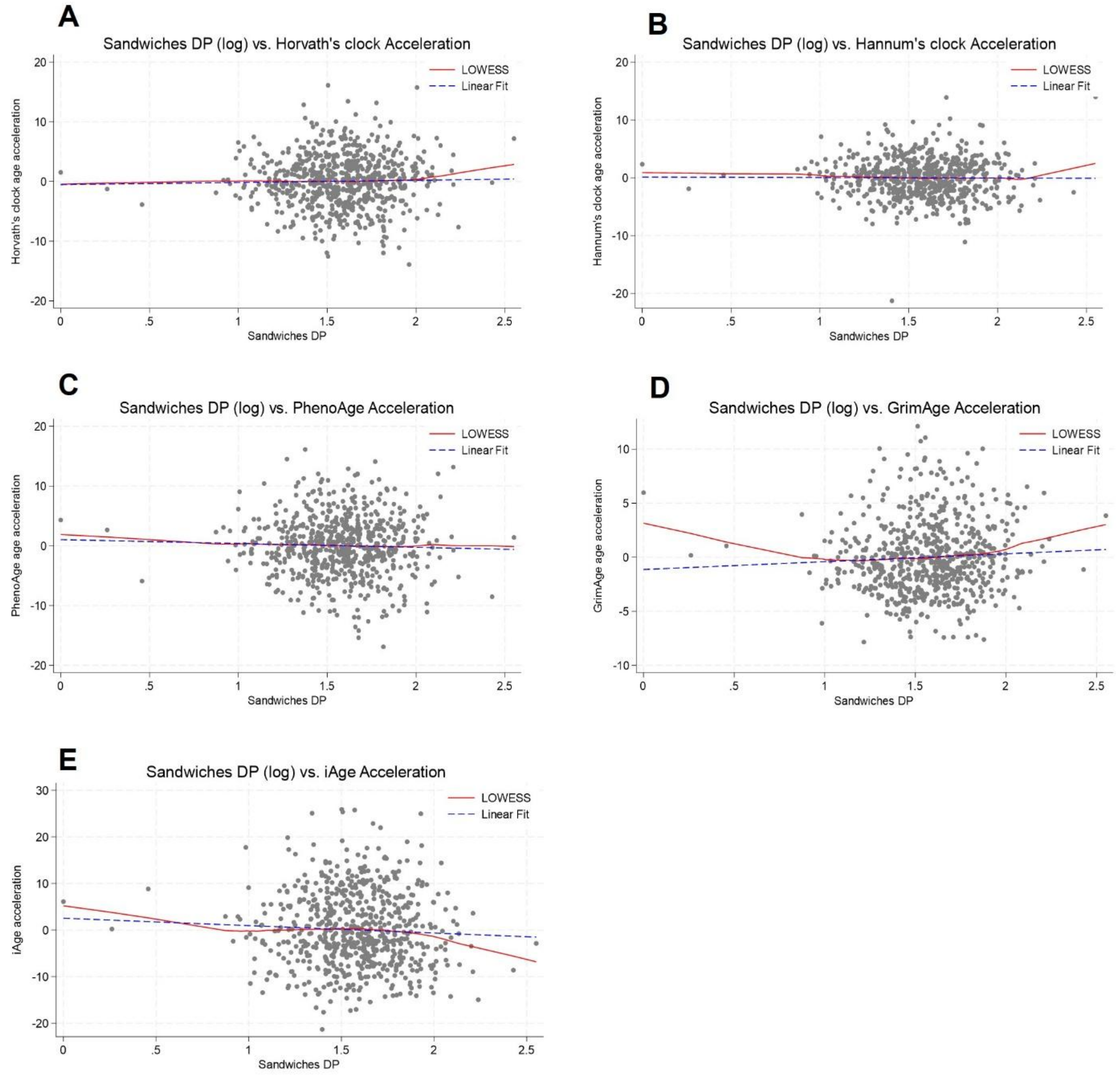

Supplementary figure 3 Scatterplots for visually inspecting linearity between the "Sandwiches" dietary pattern (DP) and biological age acceleration according to A – Horvath's clock, B – Hannum's clock, C – PhenoAge, D – GrimAge, E – iAge

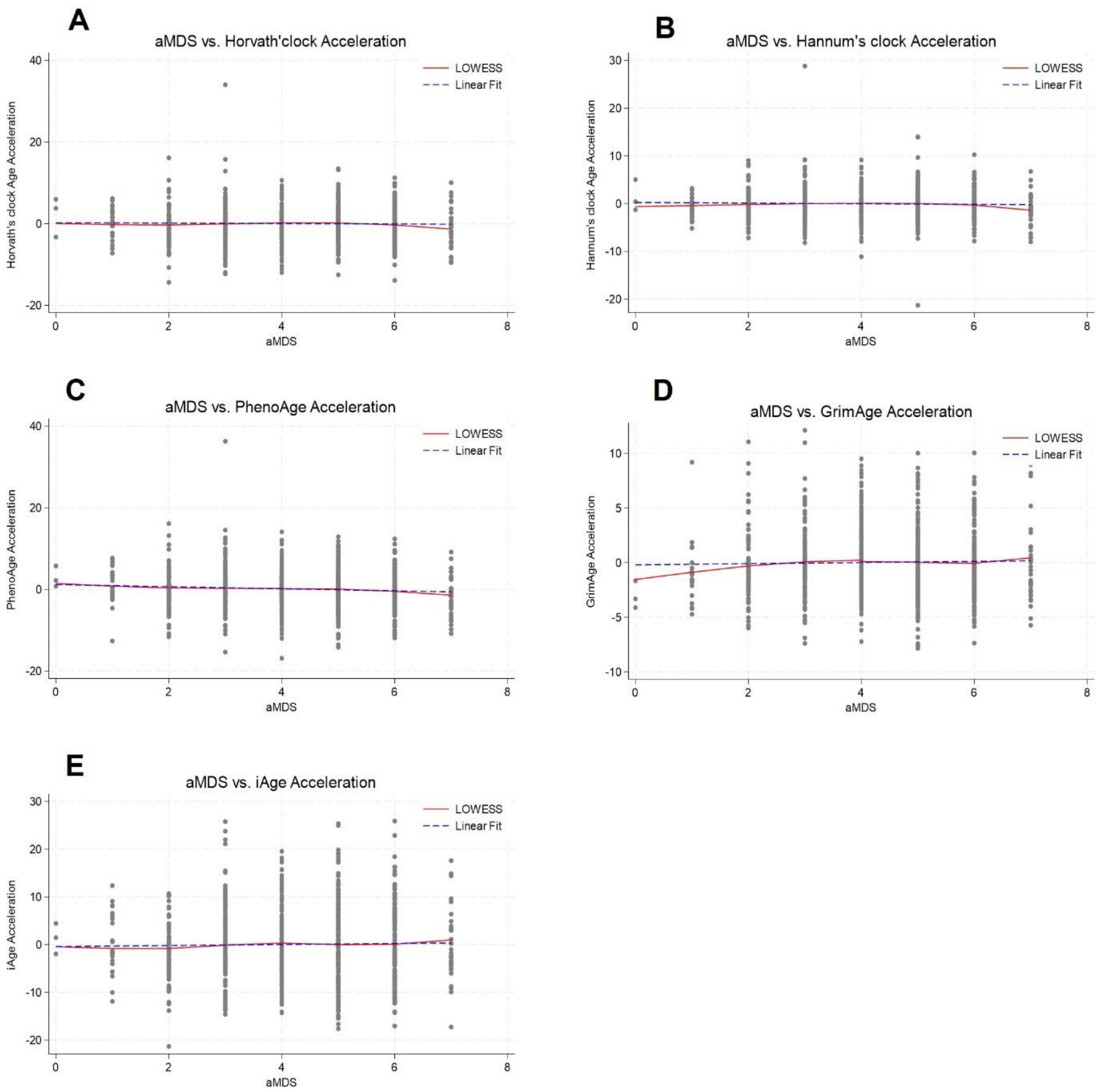

Supplementary figure 4 Scatterplots for visually inspecting linearity between the adapted Mediterranean Diet Scale (aMDS) and biological age acceleration according to A – Horvath's clock, B – Hannum's clock, C – PhenoAge, D – GrimAge, E – iAge

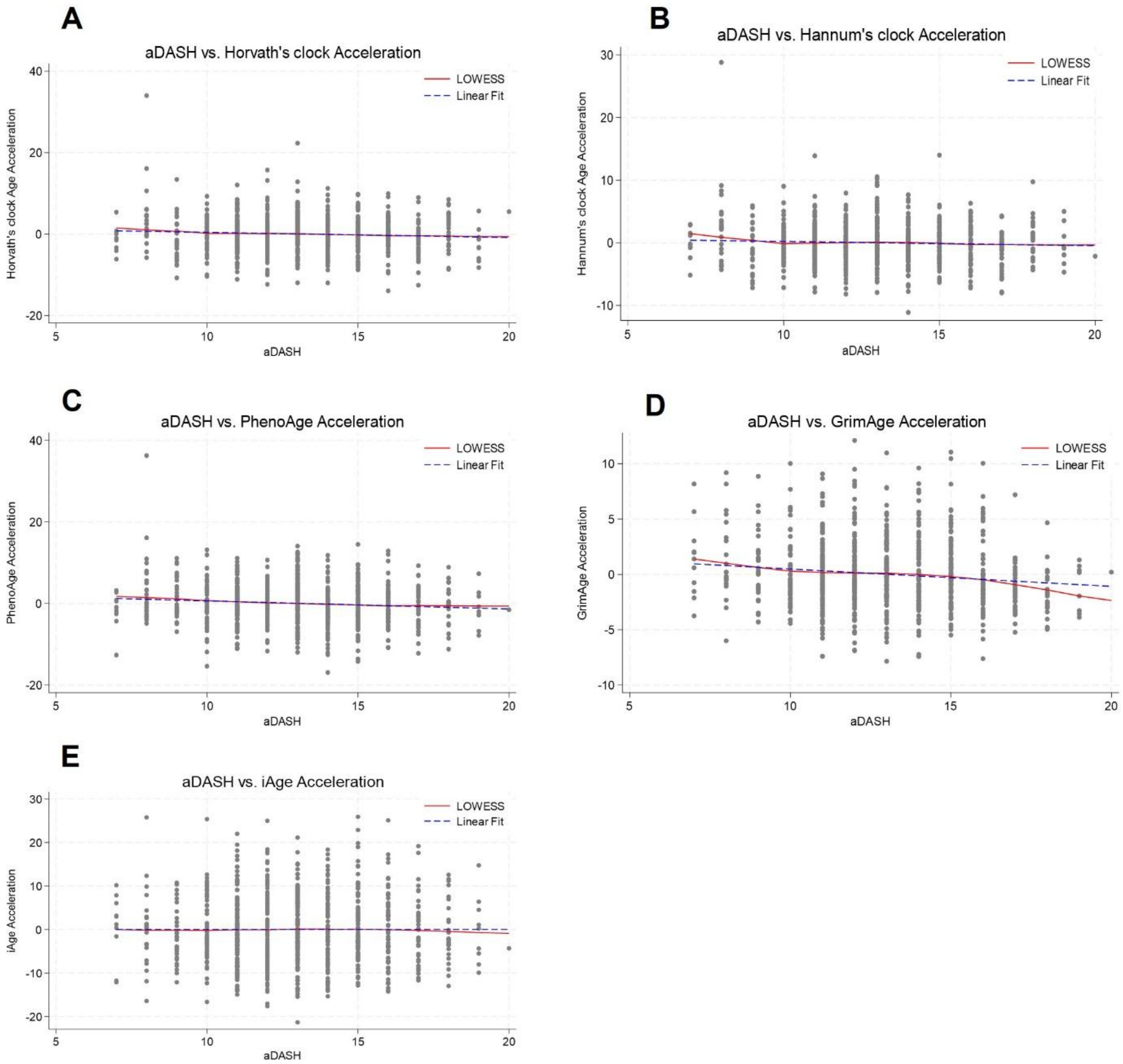

Supplementary figure 5 Scatterplots for visually inspecting linearity between the adapted DASH score (aDASH) and biological age acceleration according to A – Horvath's clock, B – Hannum's clock, C – PhenoAge, D – GrimAge, E – iAge

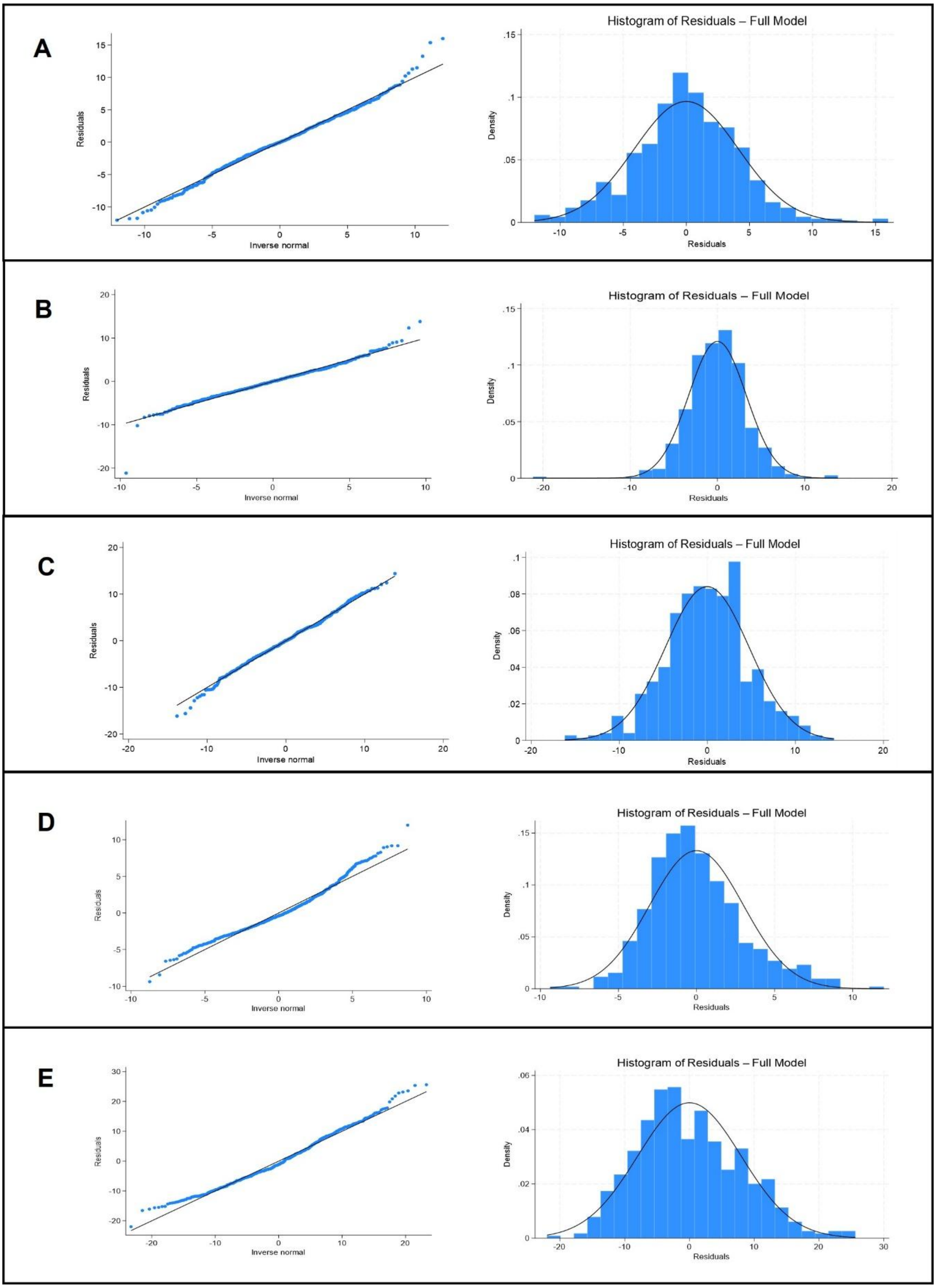

Supplementary figure 6 Quantile–quantile plots and histograms for visually inspecting the normality of the residuals from the associations between the “Plant-based” DP and biological age acceleration (Model 3) according to the A - Horvath’s clock, B - Hannun’s clock, C – PhenoAge, D -GrimAge, E - iAge

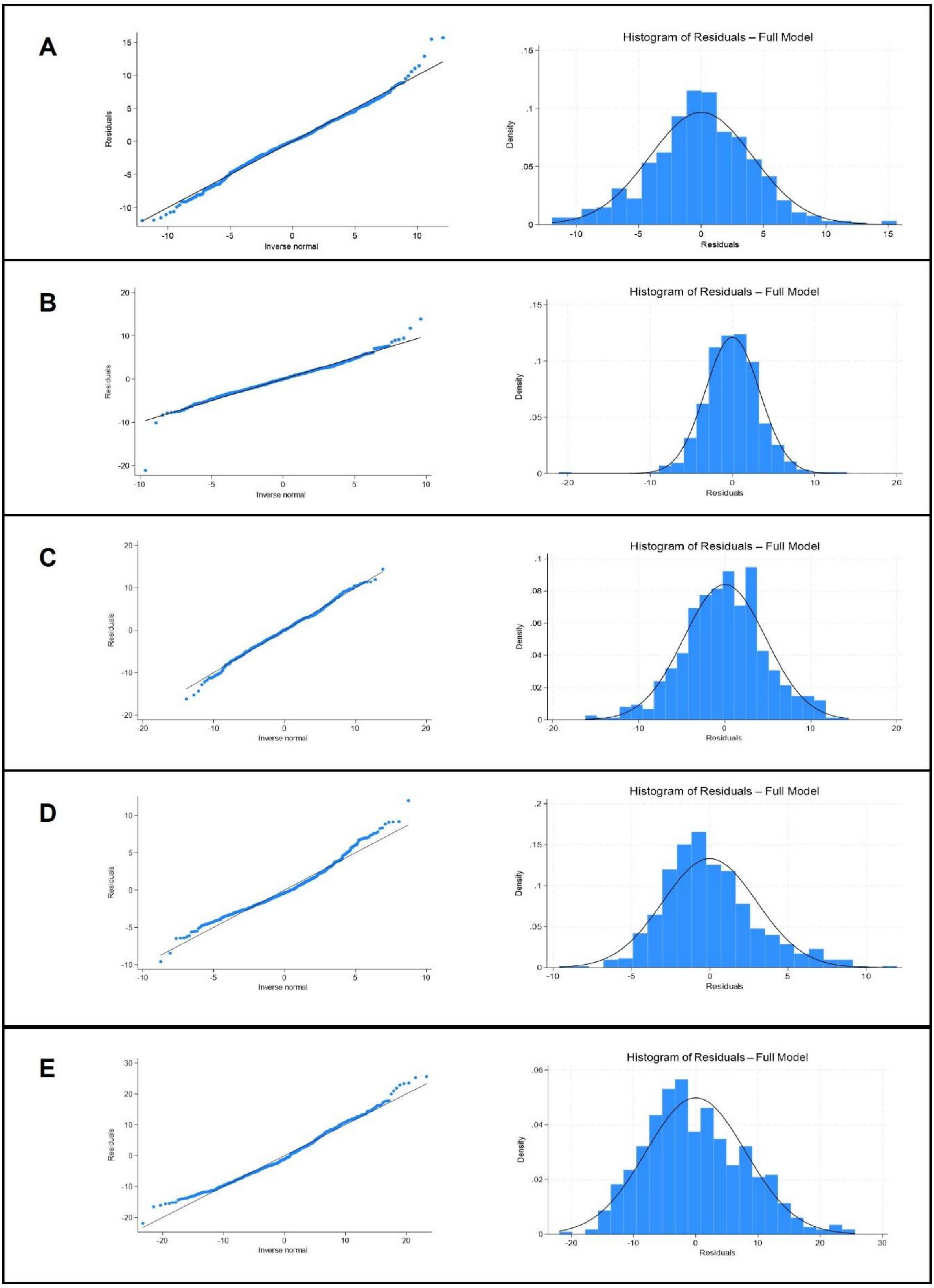

Supplementary figure 7 Quantile–quantile plots and histograms for visually inspecting the normality of the residuals from the associations between the “Sugar and fast-food” DP and biological age acceleration (Model 3) according to the A - Horvath’s clock, B - Hannun’s clock, C – PhenoAge, D – GrimAge, E - iAge

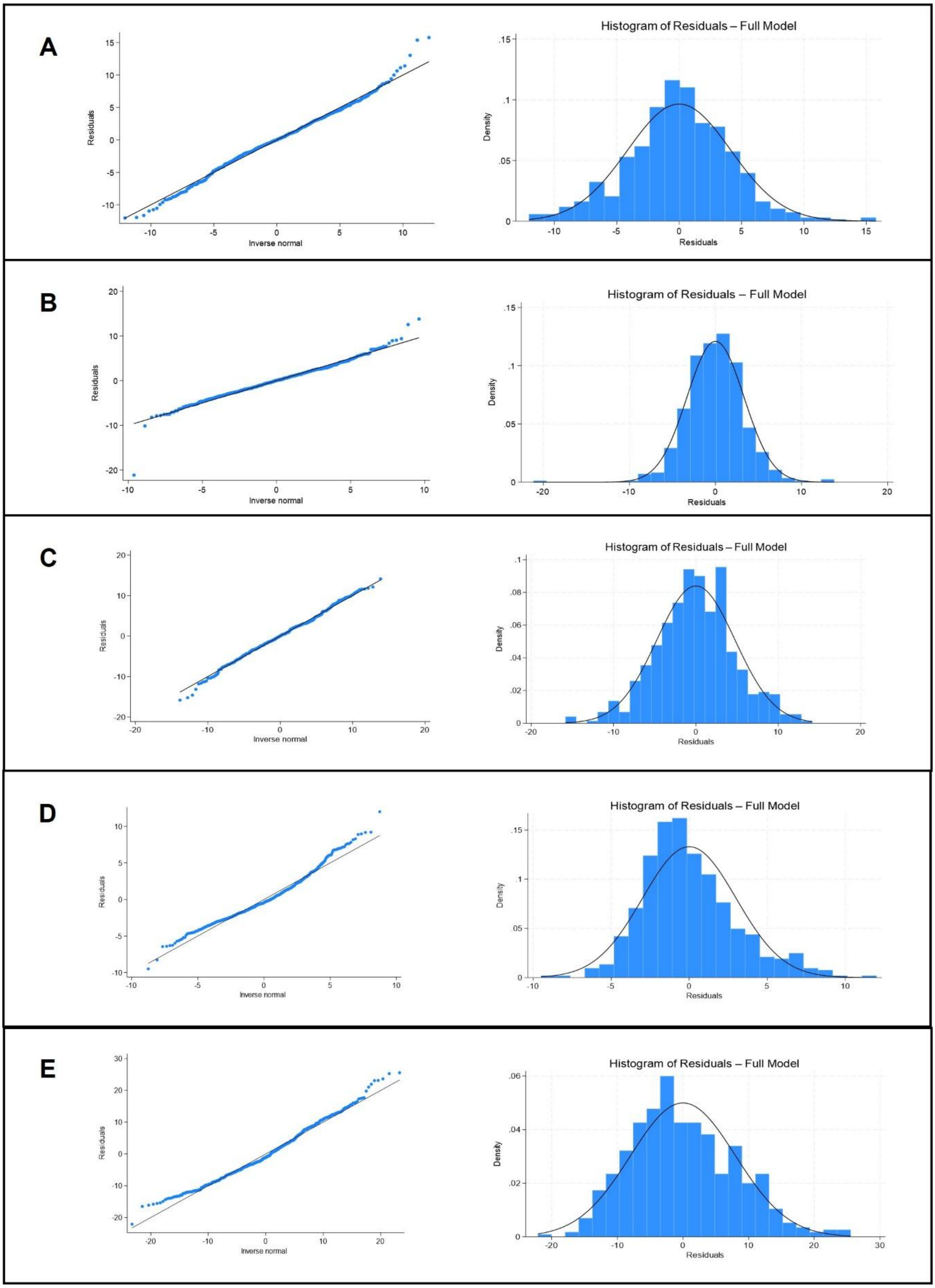

Supplementary figure 8 Quantile–quantile plots and histograms for visually inspecting the normality of the residuals from the associations between the "Sandwiches" DP and biological age acceleration (Model 3) according to the A - Horvath's clock, B - Hannun's clock, C – PhenoAge, D – GrimAge, E - iAge

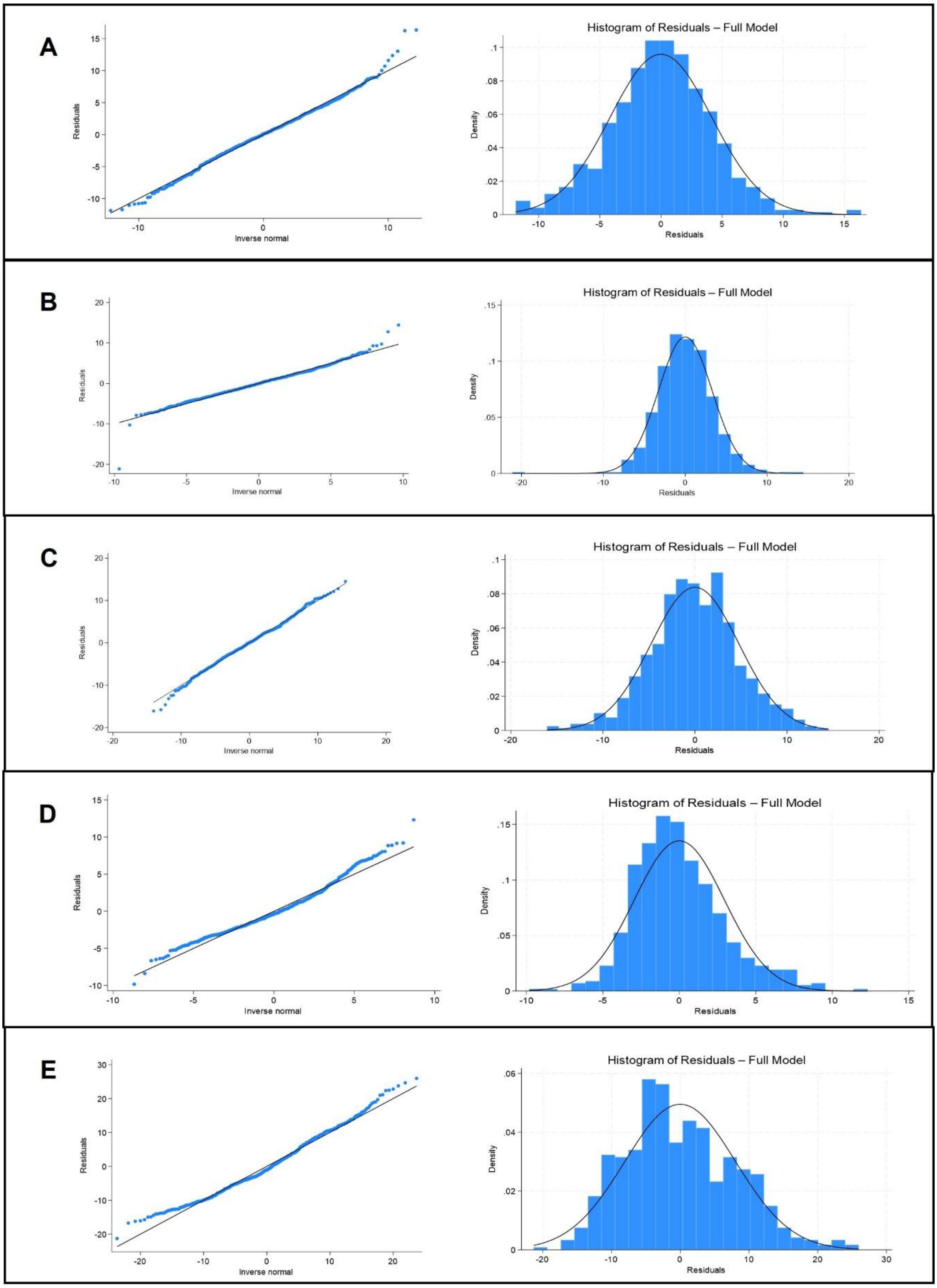

Supplementary figure 9 Quantile–quantile plots and histograms for visually inspecting the normality of the residuals from the associations between the aMDS and biological age acceleration (Model 3) according to the A - Horvath's clock, B - Hannun's clock, C – PhenoAge, D – GrimAge, E - iAge

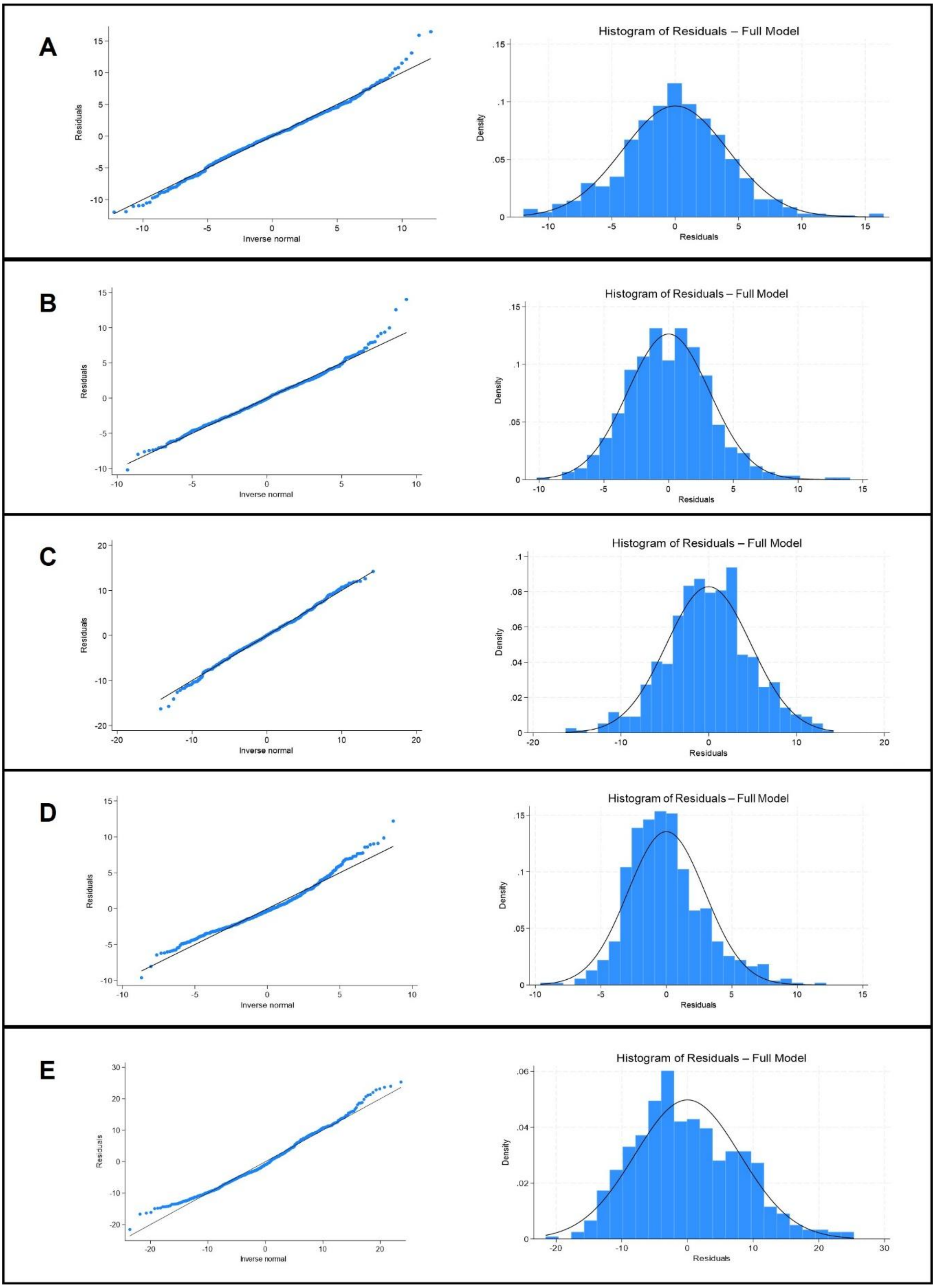

Supplementary figure 10 Quantile–quantile plots and histograms for visually inspecting the normality of the residuals from the associations between the aDASH and biological age acceleration (Model 3) according to the A - Horvath's clock, B - Hannun's clock, C – PhenoAge, D – GrimAge, E - iAge

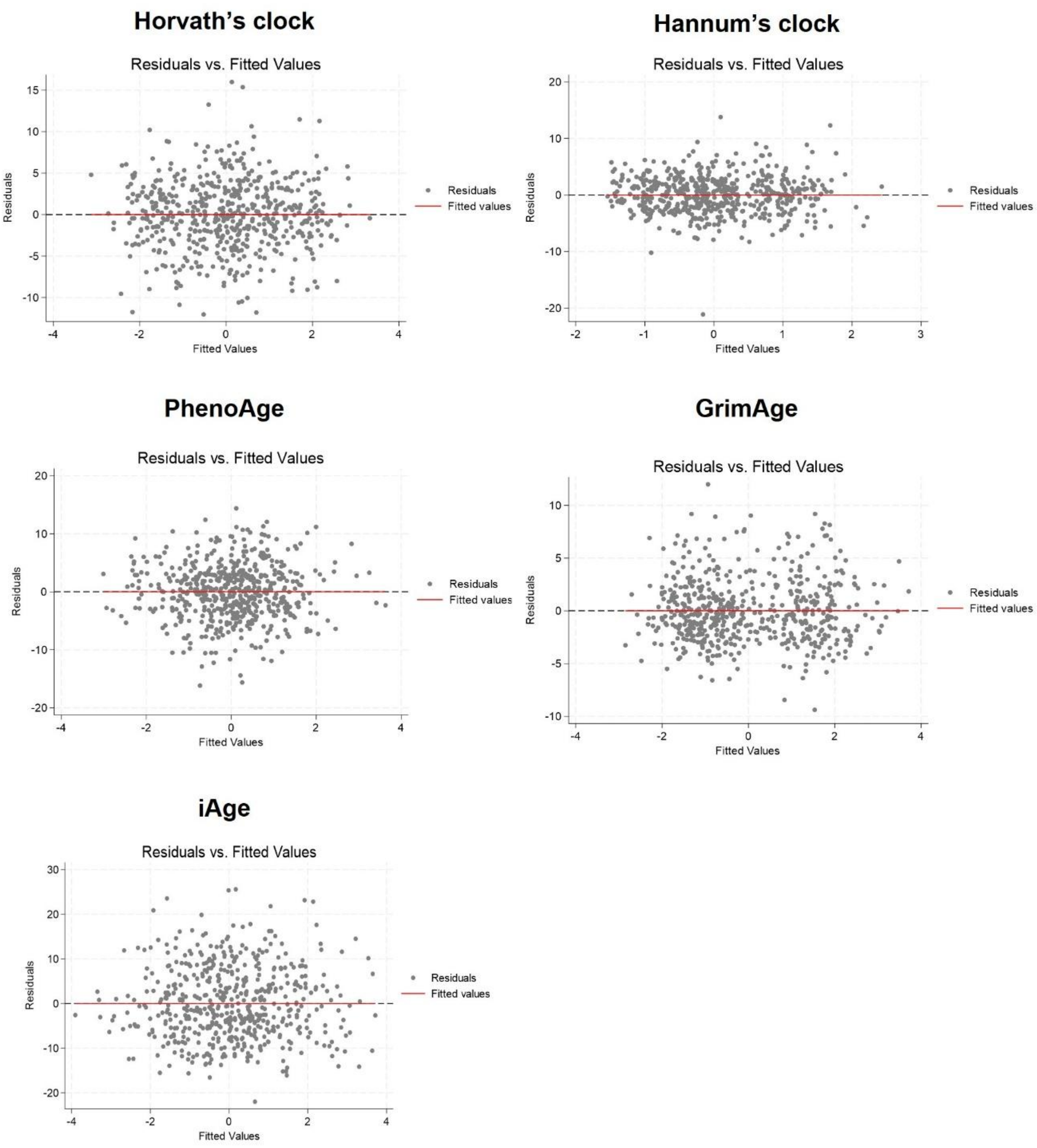

Supplementary figure 11 Scatterplots for visually inspecting homocedasticity of the full association between the "Plant-based" DP and biological age acceleration (Model 3) according to the epigenetic and inflammatory clocks

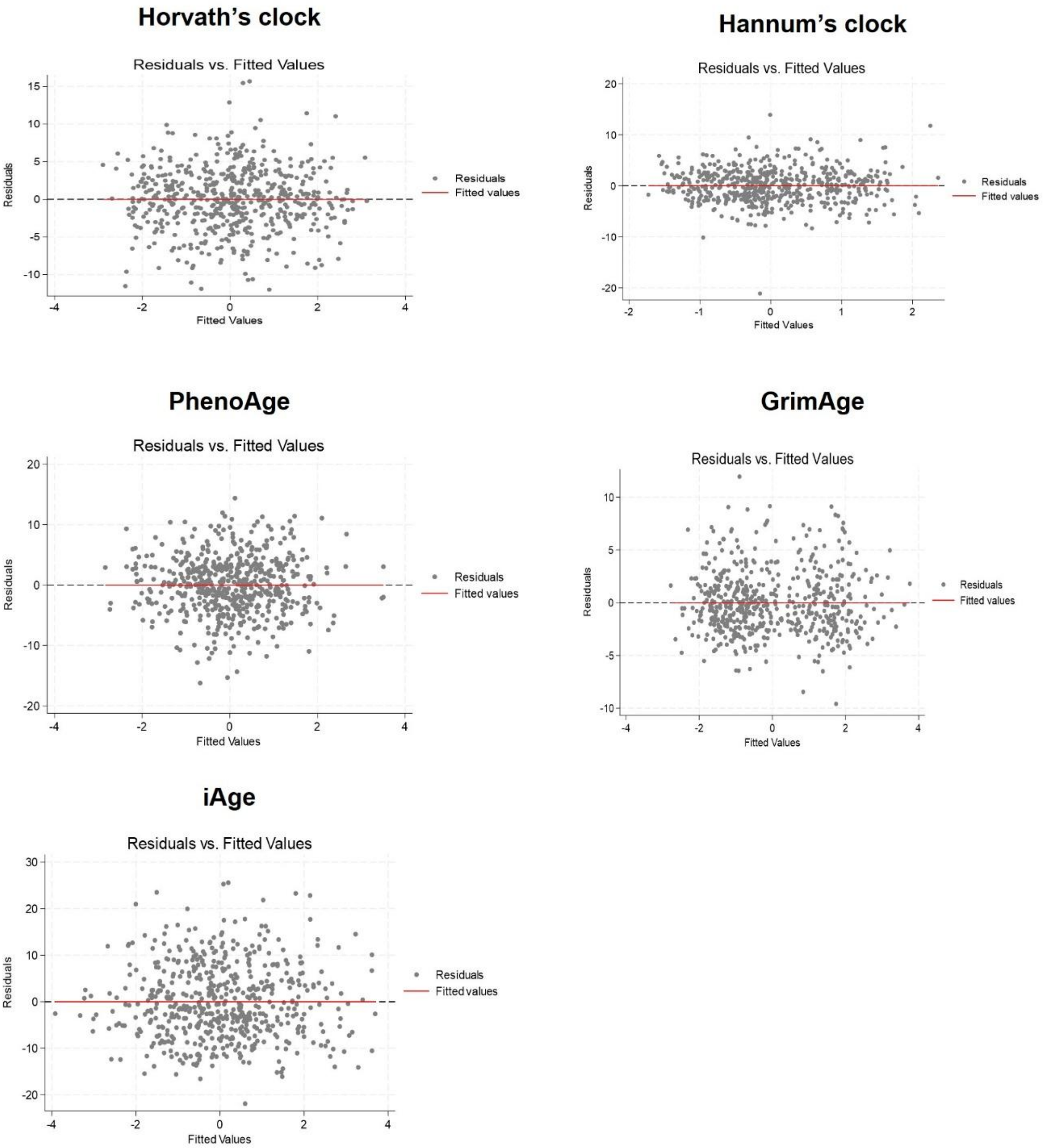

Supplementary figure 12 Scatterplots for visually inspecting homocedasticity of the full association between the “Sugar and fast-food” DP and biological age acceleration (Model 3) according to the epigenetic and inflammatory clocks

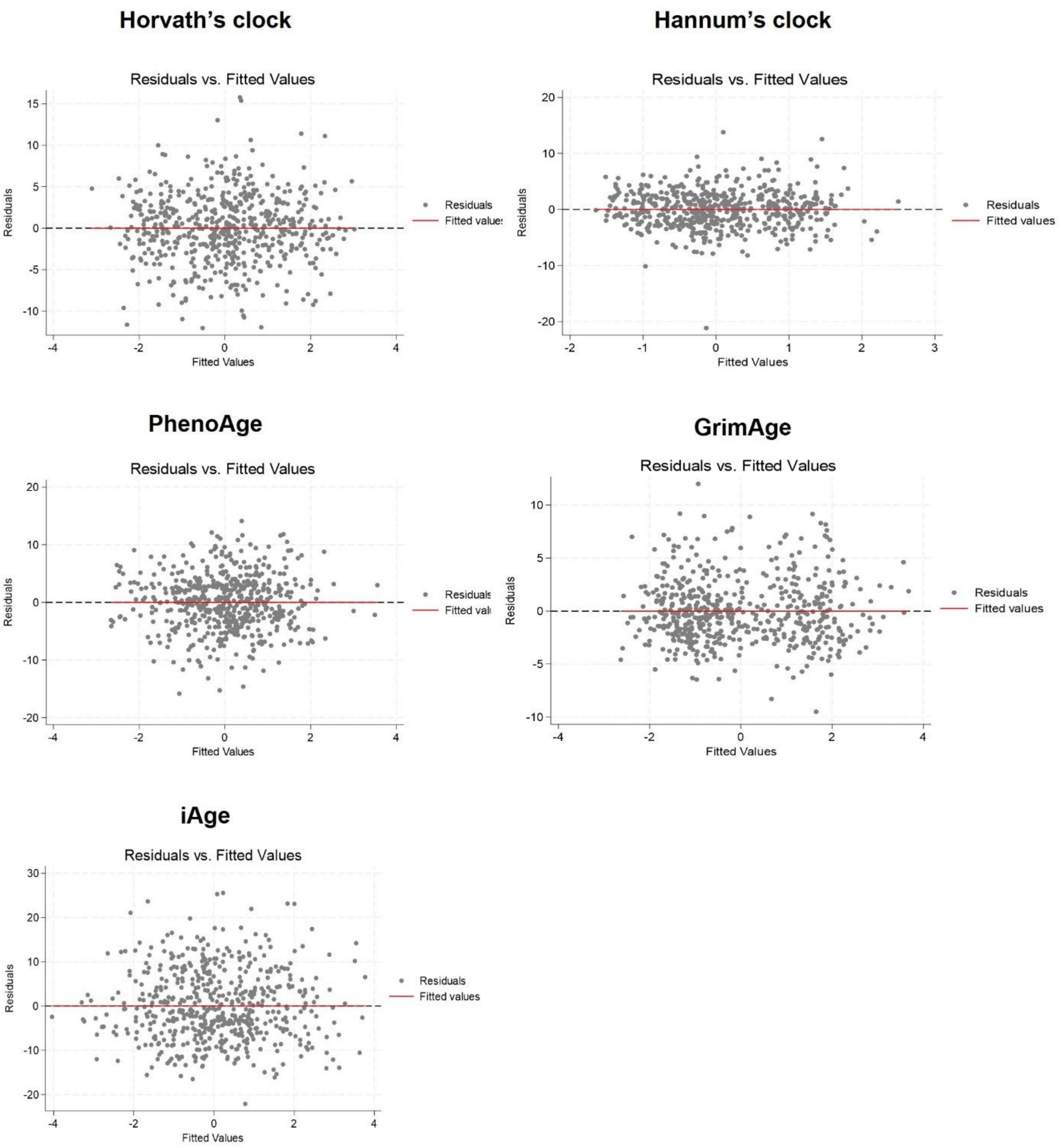

Supplementary figure 13 Scatterplots for visually inspecting homocedasticity of the full association between the "Sandwiches" DP and biological age acceleration (Model 3) according to the epigenetic and inflammatory clocks

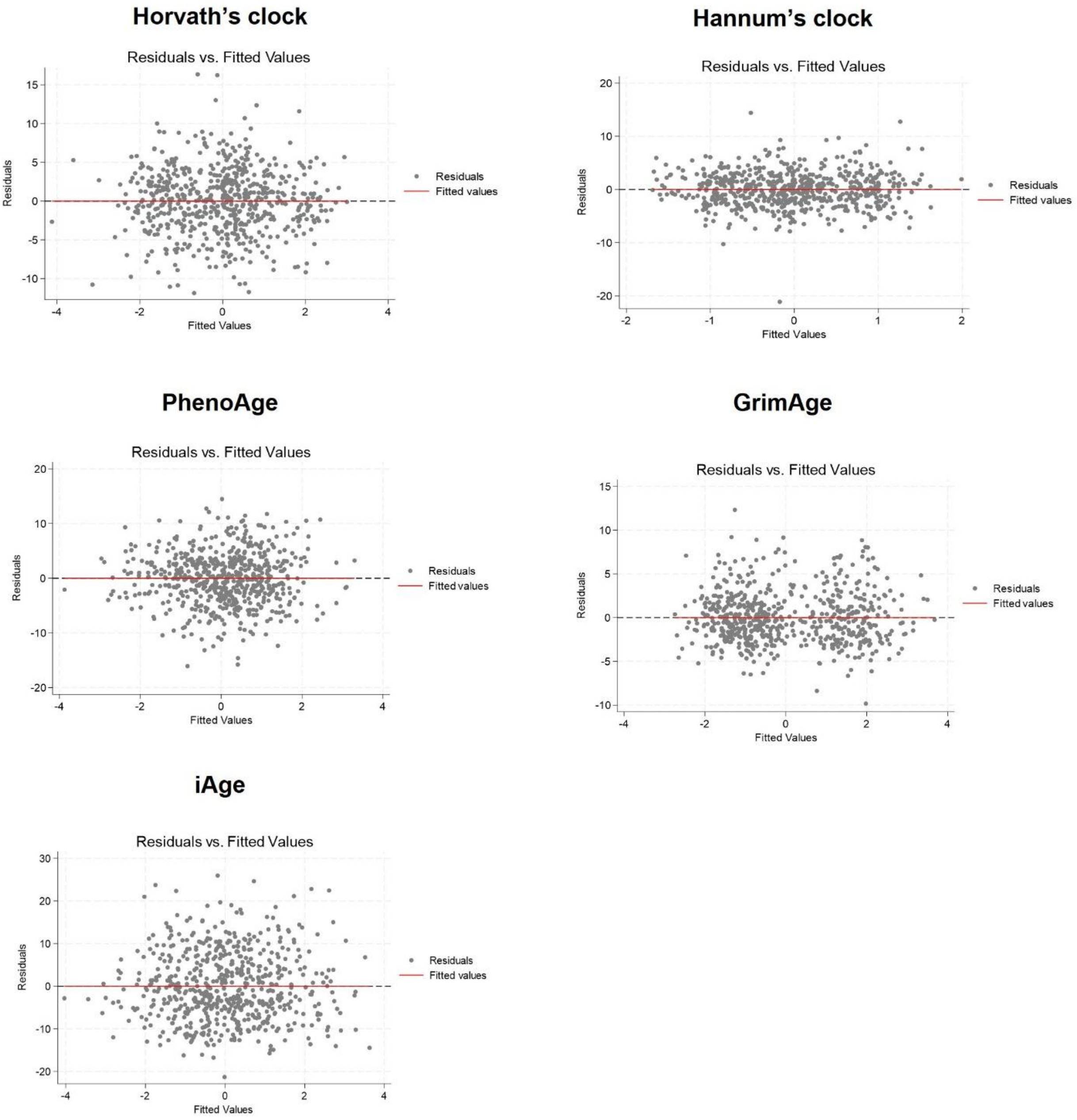

Supplementary figure 14 Scatterplots for visually inspecting homocedasticity of the full association between the aMDS and biological age acceleration (Model 3) according to the epigenetic and inflammatory clocks

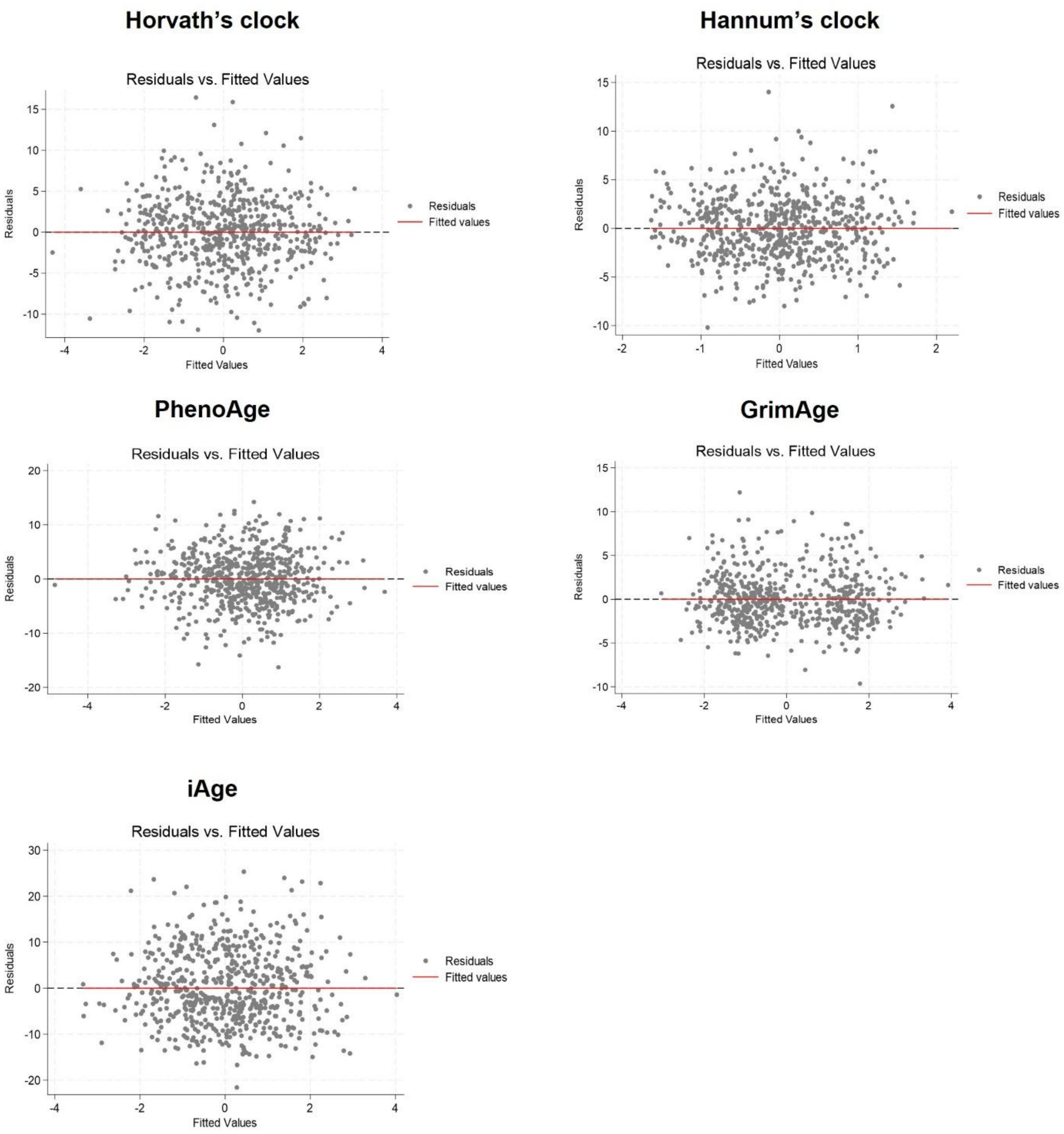

Supplementary figure 15 Scatterplots for visually inspecting homocedasticity of the full association between the aDASH and biological age acceleration (Model 3) according to the epigenetic and inflammatory clocks
