# Supplementary material for: The role of dietary patterns on epigenetic and inflammatory aging based on the INSPIRE-T study
Source: Commun Med (Lond). 2026 May 13;6:410. doi: 10.1038/s43856-026-01643-1 (PMC13396666; doi:10.1038/s43856-026-01643-1)
Supplement: Supplementary file 1 — Transparent Peer Review file [file 43856_2026_1643_MOESM1_ESM.pdf]

nature portfolio

Peer Review File

The role of dietary patterns on epigenetic and inflammatory aging based on the INSPIRE-T study

Corresponding Author: Dr Natasha Grande de França

This file contains all reviewer reports in order by version, followed by all author rebuttals in order by version.

Version 0:

Reviewer comments:

Reviewer #1

(Remarks to the Author)
The manuscript "The role of dietary patterns on epigenetic and inflammatory aging: data from the INSPIRE-T study" presents a comprehensive cross-sectional analysis examining the associations between dietary differences and epigenetic and inflammatory clocks that estimate biological aging in a large and well-characterized cohort. The study uses multiple biological clocks and mediation/moderation models to disentangle the relationships between diet, body fat, and biological aging. Overall, the research question is relevant, the analyses are detailed, and the discussion is thoughtful and mostly balanced. The work is timely, given the growing interest in nutritional determinants to ameliorate age-associated diseases in a population-wide approach. The manuscript is of high quality and should be considered for publication after minor improvements. Most issues can be addressed through clarifications in the Methods and more cautious phrasing in the Results and Discussion. The study is comprehensive, methodologically sound, and contributes meaningfully to the growing literature on dietary influences on biological aging.

- My main issue with the main text is the tendency to overinterpret findings. The results and discussion occasionally use causal phrasing ("reduces body fat," "mediated by," "associated with lower biological aging") despite the cross-sectional design. The authors could tone this down. Although the authors appropriately acknowledge this limitation later, the causal language in some sections could be avoided.
- I think that "adherence" is insufficiently defined for first-time readers.
- Please clarify how adherence to dietary patterns was specifically defined and whether these assessments were later confirmed in selected cases. As I understand, it corresponds to the ratio or score derived from the frequency of food items falling within a specific DP (e.g., aDASH, "Plant-based").
- The manuscript provides no information on long-term dietary history beyond the 4-month FFQ and no information about the general health status of the cohort.
- Importantly, since the FFQ used to assess the DP adherence, is there any information about prior changes in dietary habits? E.g., for how many years has adherence to a DP not changed? Could this be assessed from the subjective dietary FFQs?
- Was the time window of the FFQs equally distributed across major seasonal events, such as vacation periods or traditional holiday seasons?
- Since the FFQ referred to dietary intake over the prior four months, it would be important to note whether this time frame adequately reflects habitual diet. Were there any data on long-term or prior adherence (e.g., over years)?
- It would be informative if the authors could report the inflammation markers used to calculate iAge.
- The unexpected associations between "Plant-based" diets and higher iAge acceleration in younger males are acknowledged but remain speculative. While the proposed mechanisms (cortisol, antagonistic pleiotropy) are interesting, they lack empirical backing in the presented dataset. Younger males are an accepted group with more risk-related behavior and high stress at work. Could this be a factor contributing to increased iAge values?

Reviewer #2

(Remarks to the Author)
The work analysed associations between dietary patterns and epigenetic/inflammatory clocks and found that "Plant-based"

and "Sandwiches" patterns were associated with new generations of epigenetic clocks and inflammatory clock while body fat partially mediated such associations. The overall quality of this work is good while there are some parts need to be clarified.
1. In the result part: "iAge A (Year)" in eTable4, the "A" might be a typo.
2. In the discussion part: Based on eTable 5 and 6, it is interesting to see that in older adults, significances were found in PhenoAge and GrimAge while in young adults, significance was found in iAge, any explanation for that? Additionally, all the significances were from “Plant-based” and “Sandwiches” DPs but not the “Sugar and Fast food” one, which was also interesting since it has been well established that over consumptions of sugar and fast food usually lead to obesity and over-oxidation.
3. In the method part: "we considered only those with an absolute factor loading ≥0.30 (i.e., explaining ≥30% of variance) "This is an inaccurate explanation. A factor loading shows the correlation between a food group and corresponding principle component, not the variance.
4. In "Biological age acceleration":Details of DNA methylation analysis need to be included, such as preprocess, quality control, normalization etc, if applicable.
5. Still in "Biological age acceleration": Could the CpGs evaluated by the EPIC Infinitum array and passed the quality control exactly match the sites of Epigenetic clocks models used in the study? Because sometimes the latest EPIC array does not include some sites of old versions of Epigenetic clocks (e.g., Horvath and Hannum) and some sites that failed the quality control would also not be included in later calculation. The authors needed to confirm the situations mentioned above.
6. "EPIC Infinitum array", it is a typo, should be "Infinium"
7. For "Confounders": How was physical activity evaluated? Was it based on PASE or a few questions related to leisure time and transport? Also, why did the authors only focus on activities in leisure time and transport instead of daily physical activities?
8. For "Mediators": "android-to-gynoid fat ratio (i.e., fat mass in the abdominal are in relation to the hip are)" Need to revise the explanation for android-to-gynoid fat ratio.
9. For "Statistical analysis": Need to mention the constant used in DP log-transformation.

Version 1:

Reviewer comments:

Reviewer #1

(Remarks to the Author)
The authors have addressed most of my comments sufficiently. I congratulate them on the nice work and the informative and systematic analyses.

I still would like to see separate analyses of the cytokine data that underlie the iAge calculations. While the authors have done a good job explaining the rationale for using iAge and how it was measured, I believe that reporting the data individually for each cohort group and their relationship with dietary patterns could provide additional informative insights beyond the overall assessment as part of the iAge approach.

Reviewer #2

(Remarks to the Author)
The authors have addressed my comments quite well. I think the manuscript has reached the standard for publication.

Open Access This Peer Review File is licensed under a Creative Commons Attribution 4.0 International License, which permits use, sharing, adaptation, distribution and reproduction in any medium or format, as long as you give appropriate credit to the original author(s) and the source, provide a link to the Creative Commons license, and indicate if changes were made.
In cases where reviewers are anonymous, credit should be given to 'Anonymous Referee' and the source.
The images or other third party material in this Peer Review File are included in the article's Creative Commons license, unless indicated otherwise in a credit line to the material. If material is not included in the article's Creative Commons license and your intended use is not permitted by statutory regulation or exceeds the permitted use, you will need to obtain permission directly from the copyright holder.
To view a copy of this license, visit https://creativecommons.org/licenses/by/4.0/

Rebuttal Letter

Reviewer#1:
The manuscript "The role of dietary patterns on epigenetic and inflammatory aging: data from the INSPIRE-T study" presents a comprehensive cross-sectional analysis examining the associations between dietary differences and epigenetic and inflammatory clocks that estimate biological aging in a large and well-characterized cohort. The study uses multiple biological clocks and mediation/moderation models to disentangle the relationships between diet, body fat, and biological aging. Overall, the research question is relevant, the analyses are detailed, and the discussion is thoughtful and mostly balanced. The work is timely, given the growing interest in nutritional determinants to ameliorate age-associated diseases in a population-wide approach. The manuscript is of high quality and should be considered for publication after minor improvements. Most issues can be addressed through clarifications in the Methods and more cautious phrasing in the Results and Discussion. The study is comprehensive, methodologically sound, and contributes meaningfully to the growing literature on dietary influences on biological aging.

We thank the reviewer for these encouraging words! We tried our best to provide explanations for the questions raised and carefully revised the text to avoid overstating our findings.

Comment 1. My main issue with the main text is the tendency to overinterpret findings. The results and discussion occasionally use causal phrasing ("reduces body fat," "mediated by," "associated with lower biological aging") despite the cross-sectional design. The authors could tone this down. Although the authors appropriately acknowledge this limitation later, the causal language in some sections could be avoided.

We thank the reviewer for this important observation and agree that causal language should be avoided in the context of a cross-sectional design. We have carefully revised the manuscript to remove or rephrase statements that could imply causality (e.g., replacing terms such as "reduces" or "mediated by" with non-causal language such as "is associated with"). The Results and Discussion sections have been edited accordingly to ensure that interpretations remain appropriately descriptive and aligned with the study design.

Comment 2. I think that "adherence" is insufficiently defined for first-time readers.

We thank the reviewer for this valuable comment. We agree that the term "adherence" may not have been sufficiently defined for readers encountering the concept for the first time or who are not familiar with studies in epidemiological nutrition. We have now provided a clearer and more explicit definition of adherence in the context of dietary patterns in the revised manuscript. The complete clarification is presented in the subsequent response.

Comment 3. Please clarify how adherence to dietary patterns was specifically defined and whether these assessments were later confirmed in selected cases. As I understand, it corresponds to the ratio or score derived from the frequency of food items falling within a specific DP (e.g., aDASH, "Plant-based").

Thank you for this important comment. We agree that the definition of adherence in this context was not clear enough and may not have been the most appropriate. For the data-driven (a posteriori) dietary patterns, participant-specific pattern scores obtained from principal component analysis (PCA). For each participant, dietary pattern scores were calculated as weighted linear combinations of the standardized food group intakes using the scoring coefficients from the varimax-rotated solution. These scores reflect the "degree of adherence" to each identified dietary pattern, with higher values indicating greater conformity to the corresponding dietary pattern (i.e., person's intake more closely matches that pattern's combination of foods). *Although using the term "adherence" is acceptable in nutritional epidemiology, PCA scores are continuous and relative to the study sample (centered near zero and not absolute measures of intake), thus we preferred replacing "higher/lower adherence" with "higher/lower scores" in the manuscript to improve technical accuracy and avoid misinterpreting as intentional "adherence".*

For the a priori dietary patterns (Mediterranean diet and DASH), adherence was quantified using established scoring algorithms adapted to our FFQ: for the Mediterranean diet, an adapted Mediterranean Diet Scale (aMDS) was used, assigning points based on sex-specific median intakes of beneficial and detrimental food groups; for the DASH diet, an adapted DASH score (aDASH) was calculated based on seven dietary components, with beneficial components scored positively and detrimental components inversely. In these cases, as participants are compared against a predefined dietary model, "adherence" is perfectly appropriate terminology and was kept in the manuscript.

For both a posteriori and a priori dietary patterns, participants' adherence scores were treated as continuous variables (ordinal in the cases of aMDS and a DASH) in regression analyses. No additional confirmation procedure was performed, as the regression-scoring approach used in Stata is standard for deriving individual pattern scores directly from the study population. For the a priori DPs, no additional confirmation procedure was performed as well, since adherence was directly calculated from the FFQ data using a scoring method widely used in nutritional epidemiology. This clarification has been added to the Methods section.

Comment 4. The manuscript provides no information on long-term dietary history beyond the 4-month FFQ and no information about the general health status of the cohort.

We thank the reviewer for this important observation. As this is a cross-sectional study, dietary intake was assessed using a validated 4-month FFQ to capture recent habitual intake relevant to the outcomes measured at the same time point. While longer-term dietary history could provide additional context, extended recall periods are associated with greater measurement error (Worsley, 1991). We have now acknowledged the absence of lifetime dietary data as a limitation in the Discussion section.

Regarding general health status, participants were screened according to predefined inclusion/exclusion criteria to participate in the INSPIRE-T; i.e., not having a severe disease compromising life expectancy at 5 years (or at 1 year for those dependent on daily life activities) –this information is now explicit in Methods (Participants and study design). Furthermore, we collected baseline information on medical history (past/current chronic diseases and current medications taken), which shows that this population is apparently healthy (69% take a maximum of 2 medications and 48% have no medical history of any specific chronic disease) –this information is now explicitly stated in Results.

We appreciate the reviewer's suggestion, which has helped improve the transparency of the manuscript

Reference

Worsley A. Effects of varying recall periods on reported food intakes. Appetite (1991) 16(1):69-82. doi: 10.1016/0195-6663(91)90113-7.

Comment 5. Importantly, since the FFQ used to assess the DP adherence, is there any information about prior changes in dietary habits? E.g., for how many years has adherence to a DP not changed? Could this be assessed from the subjective dietary FFQs?

We thank the reviewer for this important comment. The FFQ used in this study was designed to capture habitual dietary intake over the preceding 4 months and does not assess long-term or lifetime dietary patterns. Therefore, prior changes in dietary habits or the duration of adherence to a given dietary pattern cannot be determined from the available data.

While longer retrospective assessments might provide additional historical context, extended recall periods in self-reported dietary instruments are associated with increased memory-related measurement error and misclassification (especially among older adults). For this reason, the selected timeframe was intended to balance recall accuracy with the assessment of recent habitual intake relevant to this cross-sectional analysis.

We acknowledge this limitation and have clarified it in the revised Discussion section.

Comment 6. Was the time window of the FFQs equally distributed across major seasonal events, such as vacation periods or traditional holiday seasons?

Thank you for this insightful comment, which helped strengthen our work. Indeed, when assessing habitual dietary intake of a population, it is fundamental to take into account the seasonal changes to better reflect the "real life". We examined the distribution of the FFQ application throughout the seasons, as shown in the table below. We can confirm that the application of FFQs was well balanced throughout the seasons, with a slightly higher frequency of application in winter (28%), which reflects the food intake during the autumn. There was no difference in the distribution according to the sex of the participants. For better transparency, we included this information in Table 1 in the manuscript.

Distribution of the FFQ application across the seasons

season	Total, n (%)	Males, n (%)	Females, n (%)	P*
Winter	218 (28.5)	95 (32.9)	123 (25.9)	
Spring	147 (19.2)	58 (20.1)	89 (18.7)	0.127
Summer	204 (26.7)	68 (23.5)	136 (28.6)	
Autumn	195 (25.5)	68 (23.5)	127 (26.7)	
Total	764 (100)	289 (100)	475 (100)	

*chi-squared test

Comment 7. Since the FFQ referred to dietary intake over the prior four months, it would be important to note whether this time frame adequately reflects habitual diet. Were there any data on long-term or prior adherence (e.g., over years)?

We thank the reviewer for this important comment. The FFQ assessed dietary intake over the preceding four months, a timeframe commonly used to capture recent habitual intake while minimizing recall bias. In the context of this cross-sectional analysis, this period was selected to reflect dietary exposure corresponding to the contemporaneous assessment of aging clocks. Although the Inspire-T study collects dietary intake information annually, the present analysis was restricted to baseline data because aging clock measures were only available at this timepoint. Therefore, longitudinal dietary data were not incorporated into the current study. We acknowledge that longer-term adherence data could provide additional insight. This limitation has now been clarified in the Discussion section.

Comment 8. It would be informative if the authors could report the inflammation markers used to calculate iAge.

We thank the reviewer for this observation. The iAge score was calculated using a linear regression model trained on the five inflammatory analytes that contributed most strongly to iAge prediction in the INSPIRE-T cohort: CCL11 (EOTAXIN-1), CXCL1 (GRO-α), CXCL9, IFN-γ, and TRAIL (TNF-related apoptosis-inducing ligand). In the original study describing the development of iAge, Sayed et al. (2021) reported no significant difference in prediction error between a model using these five analytes and one incorporating all 50 inflammatory markers, demonstrating that iAge can be estimated with comparable accuracy using this reduced feature set. We agree that this information was not sufficiently explicit in the original version of the manuscript. We have now revised the Methods section to clearly specify the analytes used to compute iAge and to clarify the rationale for using the reduced feature set.

Comment 9. The unexpected associations between "Plant-based" diets and higher iAge acceleration in younger males are acknowledged but remain speculative. While the proposed mechanisms (cortisol, antagonistic pleiotropy) are interesting, they lack empirical backing in the presented dataset. Younger males are an accepted group with

more risk-related behavior and high stress at work. Could this be a factor contributing to increased iAge values?

We thank the reviewer for this insightful comment. We agree that the mechanisms underlying the observed association between plant-based dietary patterns and higher iAge acceleration in younger males are not directly tested in our dataset and should be considered speculative. To clarify this in the manuscript, we have revised the Discussion to explicitly indicate the exploratory nature of our proposed explanations. We decided, however, to keep them, considering that this could prompt future investigations.

We agree that younger males tend to experience higher levels of psychosocial stress and engage in more risk-related behaviors (including smoking and alcohol consumption), both of which have been associated with immune modulation. It is suggested that psychological stress can influence inflammatory pathways, including IFN-γ–related signaling and downstream chemokines such as CXCL9, which are components of the iAge model (Ranjbaran et al., 2025). Although most stress research has focused on more common biomarkers such as IL-6 and TNF-α, broader immune network effects are plausible. However, stress-related immune activation is a potential but also unmeasured contributing factor. We tried to check whether removing any of the covariates could have an impact on this age-sex group, but this did not change the results. We also verified their number of diseases, but most of them (85%) did not report any disease. We also observed the distribution of the individuals according to iAge acceleration and the aMDS, and the Plant-based DP, and there is indeed a tendency of positive association.

However, it is important to consider that this subgroup is composed of relatively few people (n=55) and that unmeasured factors may be influencing this association. We considered this in the revised discussion.

Reference
Ranjbaran A et al. Psychological stress and social support are associated with opposing single-cell pro-inflammatory gene regulatory mechanisms in adults. bioRxiv [Preprint] (2025), 15:2025.10.14.682318. doi: 10.1101/2025.10.14.682318

Number of participants according to the age group and the sex

Age group (years)	Male	Female	Total
>=20 & <45	55	122	177
>=45 & <65	85	168	253
>=65	131	177	308
Total	271	467	738

Number of diseases reported by males according to the age group

	Number of diseases			
**Age group (years)**	**0**	**1-2**	**3**	**Total**
>=20 & <45	46 (85.2)	8 (14.8)	0 (0)	54 (100)
>=45 & <65	46 (54.8)	30 (35.7)	8 (9.5)	84 (100)
>=65	21 (16.3)	62 (48.1)	46 (35.7)	129 (100)
Total	113 (42.3)	100 (37.5)	54 (20.2)	267 (100)

Reviewer #2:

The work analysed associations between dietary patterns and epigenetic/inflammatory clocks and found that "Plant-based" and "Sandwiches" patterns were associated with new generations of epigenetic clocks and inflammatory clock while body fat partially mediated such associations. The overall quality of this work is good while there are some parts need to be clarified.

We thank the reviewer for these encouraging words! We tried our best to provide clarifications to the raised concerns and carefully revised the text.

Comment 1. In the result part: "iAge A (Year)" in eTable4, the "A" might be a typo.

Thank you for catching this typo error. The letter "A" was removed from the eTable4.

Comment 2/1. In the discussion part: Based on eTable 5 and 6, it is interesting to see that in older adults, significances were found in PhenoAge and GrimAge while in young adults, significance was found in iAge, any explanation for that?

We thank the reviewer for this insightful comment. Although our data limit providing an empirical explanation, we can speculate that the observed differences may reflect the distinct biological processes captured by the different clocks. iAge is primarily an inflammatory clock and may be more sensitive to dynamic immune states. In younger adults, chronic low-grade inflammation may be more variable and influenced by lifestyle or stress-related factors rather than accumulated age-related damage, potentially allowing diet-related associations to emerge more readily. In contrast, PhenoAge and GrimAge are DNA methylation–based, multi-system clocks that reflect cumulative physiological dysregulation and long-term exposures. In older adults, these clocks may better capture the integrated effects of lifelong dietary patterns and metabolic stress. Younger adults typically exhibit lower baseline acceleration and reduced variability in methylation-based clocks, which may limit statistical power to detect associations in this age group. Taken together, these findings suggest that iAge may detect diet-associated immune changes earlier in life, whereas PhenoAge and GrimAge may require longer-term exposure to manifest measurable associations.

Comment 2/2. Additionally, all the significances were from "Plant-based" and "Sandwiches" DPs but not the "Sugar and Fast food" one, which was also interesting since it has been well established that over consumptions of sugar and fast food usually lead to obesity and over-oxidation.

We thank the reviewer for this thoughtful observation. We agree that a dietary pattern characterized by higher intake of sugar and fast food would be expected to associate with adverse biological aging outcomes. In our cohort, this pattern explained a slightly smaller proportion of the variability in dietary intake (9.3%) compared with the "Plant-based"(10%) and "Sandwiches" DPs (9.5%), which may have reduced its relative statistical power to detect associations.

Importantly, we did observe a positive association between higher adherence to the "Sugar and Fast food" pattern and GrimAge acceleration in the crude model ($\beta = 0.98$, $p = 0.029$). However, this association lost statistical significance after adjustment for covariates. This attenuation suggests that the relationship may be partly confounded by factors such as BMI in the model. Notably, for most biological clocks (except the first-generation Hannum clock), the direction of association remained positive, indicating a consistent trend toward higher biological age acceleration, although these did not reach statistical significance.

Taken together, our findings suggest that while the "Sugar and Fast food" pattern shows a directionally consistent association with accelerated biological aging, its effect may be more modest in this population, partly explained by downstream metabolic factors or limited by statistical power.

Comment 3. In the method part: "we considered only those with an absolute factor loading ≥0.30 (i.e., explaining ≥30% of variance) "This is an inaccurate explanation. A factor loading shows the correlation between a food group and corresponding principle component, not the variance.

We thank the reviewer for this important comment. We agree that our previous statement was inaccurate. Factor loadings indicate the strength and direction of the correlation between each food group and the corresponding principal component (dietary pattern), *not the proportion of variance explained. In our analysis, we considered factor loadings ≥0.30 as the variables that meaningfully contribute to each factor (i.e., represent the main features of the dietary pattern)*. *The manuscript has been updated to reflect this accurate explanation.*

Comment 4. In "Biological age acceleration":Details of DNA methylation analysis need to be included, such as preprocess, quality control, normalization etc, if applicable.

We thank the reviewer for this important comment. We have revised the Methods section to provide additional details regarding preprocessing, normalization, and imputation procedures used for DNA methylation-based age estimation.

DNA methylation was measured using the Illumina Infinium MethylationEPIC BeadChip (EPIC v1). Raw intensity data were processed in Partek® Genomics Suite® to derive methylation β-

values ranging from 0 (fully unmethylated) to 1 (fully methylated). No additional normalization was applied within Partek before epigenetic clock computation.

Horvath's clock (2013), Hannum's clock, and PhenoAge (Levine) were estimated using the methylclock R package. Normalization was enabled (normalize = TRUE), which applies the BMIQ-based normalization procedure implemented in the package. When required, missing CpG were handle using the package's t default internal imputation procedure (fastImp = FALSE).

GrimAge was calculated following the original method described by Lu et al. Normalization was enabled in accordance with the recommended settings (BMIQ-based normalization), and k-nearest neighbor (kNN) imputation was applied to handle missing CpGs.

These methodological details have now been incorporated into the revised manuscript.

Comment 5. Still in "Biological age acceleration": Could the CpGs evaluated by the EPIC Infinitum array and passed the quality control exactly match the sites of Epigenetic clocks models used in the study? Because sometimes the latest EPIC array does not include some sites of old versions of Epigenetic clocks (e.g., Horvath and Hannum) and some sites that failed the quality control would also not be included in later calculation. The authors needed to confirm the situations mentioned above.

We thank the reviewer for raising this important methodological point. DNA methylation profiling was performed using the Illumina EPIC v1 array, which covers the vast majority of CpGs included in first-generation epigenetic clocks.

For Horvath's, Hannum's, and PhenoAge clocks, compatibility between the EPIC v1 platform and the required CpG sets was handled through the methylclock package. The DNAmAge function evaluates the proportion of clock-specific CpGs present in the dataset, with a minimum required proportion of 80% (min.perc = 0.8 by default). This threshold was satisfied for all samples included in our analyses. When CpGs required for a given clock were not available on the EPIC array or contained missing values, they were handled using the package's internal imputation procedure.

For GrimAge, the algorithm was applied according to the original description by Lu et al. Missing CpGs were handled using the kNN-based imputation procedure implemented in the validated computational pipeline. Overall, the proportion of missing CpGs was minimal and did not materially affect the computation of epigenetic age estimates. All clocks were calculated using validated implementations consistent with their original publications.

Comment 6. "EPIC Infinitum array", it is a typo, should be "Infinium"

Thank you for pointing out this typo, which has now been corrected in the revised manuscript.

Comment 7. For "Confounders": How was physical activity evaluated? Was it based on PASE or a few questions related to leisure time and transport? Also, why did the authors only focus on activities in leisure time and transport instead of daily physical activities?

We thank the reviewer for raising this issue. In the INSPIRE-T cohort, physical activity was assessed using the long version of the International Physical Activity Questionnaire (IPAQ). Although the reference was previously provided, we acknowledge that this was not clearly described in the Methods section. The manuscript has now been revised to explicitly state it.

We restricted our analyses to leisure-time and transport-related physical activity for two main reasons. First, limiting the analysis to these domains minimized data loss due to incomplete responses across all IPAQ domains. Second, and more importantly, leisure-time and transport-related physical activity have consistently demonstrated stronger and more robust associations with aging processes and age-related diseases compared with occupational activity or total physical activity. Previous studies have shown that leisure-time—but not occupational—physical activity is associated with a more favorable cardiovascular profile and lower cardiovascular risk (Koenig et al., 1997; Sofi et al., 2007; Byambasukh et al., 2020). Moreover, recent evidence indicates that leisure-time moderate-to-vigorous physical activity exerts a greater protective effect on biological age acceleration than total physical activity (Zhou et al., 2025). Therefore, we focused on these domains as they are more closely linked to biological aging and health outcomes relevant to our study.

References
Koenig W et al. Leisure-Time Physical Activity but Not Work-Related Physical Activity Is Associated With Decreased Plasma Viscosity: Results From a Large Population Sample. Circulation (1997). *https://doi.org/10.1161/01.CIR.95.2.335*

Sofi F et al. Leisure time but not occupational physical activity significantly affects cardiovascular risk factors in an adult population. Eur J Clin Investig (2007). https://doi.org/10.1111/j.1365-2362.2007.01884.x

Byambasukh O, Snieder H, Corpelejin E. Relation Between Leisure Time, Commuting, and Occupational Physical Activity With Blood Pressure in 125 402 Adults: The Lifelines Cohort. J Am Heart Assoc (2020). https://doi.org/10.1161/JAHA.119.014313

Zhou J et al. The impact of behavioral patterns on accelerated aging in the UK biobank. Aging Clin Exp Res (2025). https://doi.org/10.1007/s40520-025-03074-9

Comment 8. For "Mediators": "android-to-gynoid fat ratio (i.e., fat mass in the abdominal are in relation to the hip are)" Need to revise the explanation for android-to-gynoid fat ratio.

We thank the reviewer for catching this typo, which prevented a correct understanding of the text. The explanation for the android-gynoid fat ratio was updated in the manuscript as follows:
"…android-to-gynoid fat ratio (i.e., the ratio of fat around the trunk/abdominal area to the fat around the femoral-hip area)…"

Comment 9. For "Statistical analysis": Need to mention the constant used in DP log-transformation.

Thank you for this comment, which helps improve the transparency and reproducibility of our work. The dietary pattern (DP) scores were not normally distributed and included negative values. Because linear regression assumes normally distributed residuals (rather than normally distributed predictors), we examined model residuals and observed deviations from normality when using the untransformed DP scores. Therefore, the DP scores were natural log-transformed to improve model fit and residual distribution. To accommodate negative values, a constant equal to the absolute value of the minimum observed score plus 1 was added to each DP before transformation (|minimum DP score| + 1). The constants were 3.75 for the "Plant-based" DP, 4.10 for the "Sugar and fast-food" DP, and 4.99 for the "Sandwiches" DP. This clarification has now been added to the Methods section.

REBUTTAL LETTER

REVIEWER #1 (REMARKS TO THE AUTHOR):

The authors have addressed most of my comments sufficiently. I congratulate them on the nice work and the informative and systematic analyses.
I still would like to see separate analyses of the cytokine data that underlie the iAge calculations. While the authors have done a good job explaining the rationale for using iAge and how it was measured, I believe that reporting the data individually for each cohort group and their relationship with dietary patterns could provide additional informative insights beyond the overall assessment as part of the iAge approach.

The authors appreciate that the responses provided were considered of sufficient quality to satisfy the reviewer's concerns, which were truly helpful in improving our work!

The authors also thank the reviewer for this very pertinent suggestion to explore the cytokines underlying iAge. We agree that this would complement our findings and could help to explain some unexpected associations with this biological clock more empirically. Unfortunately, however, we do not have access to such detailed information. The iAge prediction from the INSPIRE-T cohort sera was obtained through a collaboration with the research group that developed this clock (doi:10.1038/s43587-021-00082-y), led by Dr. David Furman (Buck Institute for Research on Aging; Stanford School of Medicine). Only the results for iAge as a whole were provided, not for each component individually. We regret that we cannot fulfill this last request and hope that the reviewer understands.

REVIEWER #2 (REMARKS TO THE AUTHOR):

The authors have addressed my comments quite well. I think the manuscript has reached the standard for publication.

The authors thank the reviewer for the helpful comments, which were essential in improving our work.
